# Proline is increased in allergic asthma and promotes airway remodeling

**DOI:** 10.1172/jci.insight.167395

**Published:** 2023-08-22

**Authors:** Tingting Xu, Zhenzhen Wu, Qi Yuan, Xijie Zhang, Yanan Liu, Chaojie Wu, Meijuan Song, Jingjing Wu, Jingxian Jiang, Zhengxia Wang, Zhongqi Chen, Mingshun Zhang, Mao Huang, Ningfei Ji

**Affiliations:** 1 Department of Respiratory and Critical Care Medicine, The First Affiliated Hospital of Nanjing Medical University, Nanjing, China.; 2 Department of Respiratory and Critical Care Medicine, The Affiliated Hospital of Xuzhou Medical University, Xuzhou, China.; 3 NHC Key Laboratory of Antibody Technique, Jiangsu Province Engineering Research Center of Antibody Drug, Department of Immunology, Nanjing Medical University, Nanjing, China.

**Keywords:** Metabolism, Pulmonology, Amino acid metabolism, Asthma

## Abstract

Proline and its synthesis enzyme pyrroline-5-carboxylate reductase 1 (PYCR1) are implicated in epithelial-mesenchymal transition (EMT), yet how proline and PYCR1 function in allergic asthmatic airway remodeling via EMT has not yet been addressed to our knowledge. In the present study, increased levels of plasma proline and PYCR1 were observed in patients with asthma. Similarly, proline and PYCR1 in lung tissues were high in a murine allergic asthma model induced by house dust mites (HDMs). *Pycr1* knockout decreased proline in lung tissues, with reduced airway remodeling and EMT. Mechanistically, loss of *Pycr1* restrained HDM-induced EMT by modulating mitochondrial fission, metabolic reprogramming, and the AKT/mTORC1 and WNT3a/β-catenin signaling pathways in airway epithelial cells. Therapeutic inhibition of PYCR1 in wild-type mice disrupted HDM-induced airway inflammation and remodeling. Deprivation of exogenous proline relieved HDM-induced airway remodeling to some extent. Collectively, this study illuminates that proline and PYCR1 involved with airway remodeling in allergic asthma could be viable targets for asthma treatment.

## Graphical abstract

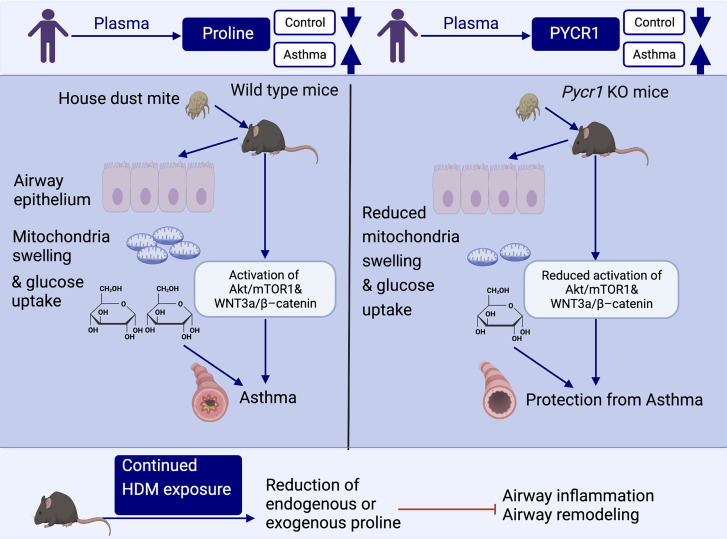

## Introduction

Asthma is a common noncommunicable disease (1, 2) with a complex etiology (3). The prevalence and burden of asthma are rapidly rising, especially in low-income and middle-income countries (4, 5). Exposure to environmental allergens such as house dust mites (HDMs) is associated with an increased risk of developing asthma, which might cause chronic allergic asthma characterized by airway remodeling (6, 7). Airway remodeling is a long-term process of changes in airway structures, resulting in disruption of the epithelial barrier, epithelial cell state change, subepithelial fibrosis, collagen deposition, and smooth muscle hyperplasia (8). Subsequently, this process leads to stenosis and obstruction of the small airways and reduced lung compliance, accounting for increased exacerbations and mortality of asthma (9). Currently, the majority of asthma cases are well controlled by recommended therapies, such as inhaled corticosteroids and β_2_-adrenergic agonists. These treatments primarily have antiinflammatory effects, whereas they are less effective for treating airway remodeling (10, 11). Due to airway remodeling and constriction, approximately 10% of patients with asthma still experience poor control after receiving medications (12). Thus, to prevent asthmatic airway remodeling and relieve disease severity in the early phases, a clear understanding of the cellular and molecular mechanisms of airway remodeling is critical for the discovery of novel targets.

Recently, it has been shown that airway remodeling is associated with epithelial cell state changes, such as epithelial-mesenchymal transition (EMT), to enhance fibrosis (3). EMT is defined as a multifaceted status in cellular phenotypes characterized by gradual loss of epithelial features, reduced cell-cell adhesive properties, and acquisition of mesenchymal features, which has been implicated in fibrosis, angiogenesis, wound healing, cancer metastasis, and chronic asthma (13–16). During this process, epithelial markers, including E-cadherin, are suppressed, while mesenchymal markers, such as α–smooth muscle actin (α-SMA) and vimentin, and fibroblast proliferation transcription factors, such as Snail and Twist, are upregulated (13). Allergens trigger the airway epithelium to secrete growth factors, i.e., transforming growth factor-beta (TGF-β) superfamily members, matrix metalloproteinases, and epidermal growth factor (EGF), ultimately causing mesenchymal cell status, airway thickening, subepithelial fibrosis, and collagen deposition in asthma (3).

Previous studies have implicated amino acids in inflammation and remodeling in asthma, such as glutamine, l-tyrosine, methionine, l-arginine, l-citrulline, hydroxyproline, and proline (17–23), among which hydroxyproline and proline are the main collagen components. The proline metabolism pathway has been well characterized for its multiple roles in promoting tumorigenesis and cancer progression (24). These studies primarily emphasized the role of proline in tumor metabolism without providing mechanistic insights into its role in the asthmatic remodeling process. Notably, pyrroline-5-carboxylate reductase 1 (PYCR1) is a crucial enzyme that catalyzes the synthesis of proline in the last step (25). PYCR1 is involved in various cell behaviors, including cell proliferation, cell migration, EMT, and regulation of metabolic pathways (26–28). A previous study indicated that the mRNA level of *Pycr1* was upregulated in the airway epithelium of an HDM-induced asthma model (29). However, there is a lack of detailed information on whether and how PYCR1 regulates EMT-mediated airway remodeling through proline production in asthma. This led us to explore whether PYCR1 and proline affect airway remodeling in an HDM-driven model of asthma.

In this study, we demonstrate that proline and PYCR1 are elevated in plasma in patients with asthma compared with healthy controls. In addition, *Pycr1* is vital for the development of HDM-induced allergic asthma in mice. Mice lacking *Pycr1* failed to develop a mutant allergic response to HDM. Our data indicate that *Pycr1* modulates mitochondrial morphology and cellular metabolism in airway epithelial cells and regulates the WNT3a/β-catenin and AKT/mTORC1 signaling pathways. In addition, deprivation of exogenous proline or inhibition of PYCR1 activity could suppress key asthma features, especially EMT-mediated remodeling. Given that restraint of EMT is seen in PYCR1-inhibited or proline-free diet mice, PYCR1 and proline may be potential drug targets for airway remodeling therapy in asthma.

## Results

### Plasma proline and PYCR1 are increased in patients with asthma.

**Figure 1:**
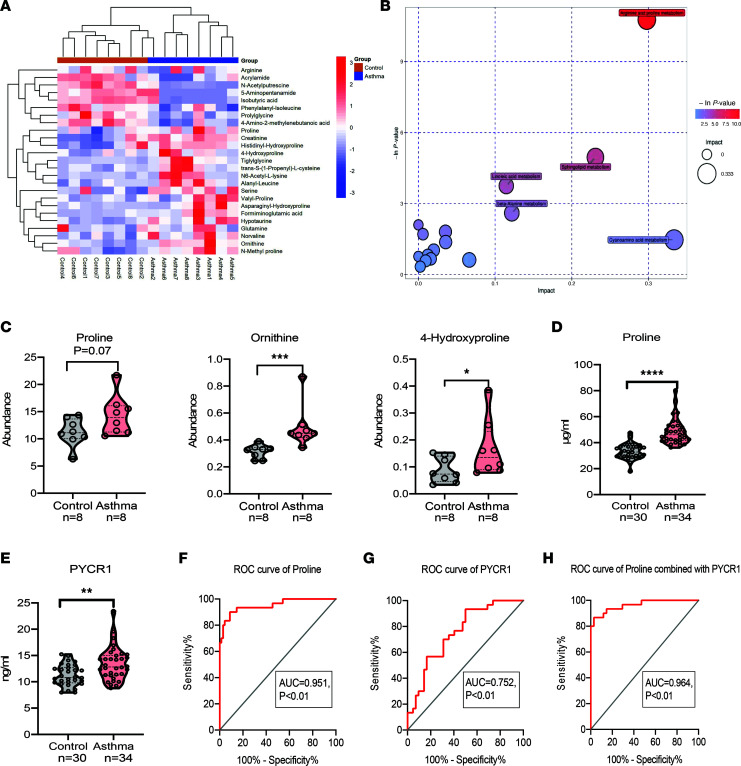
Metabolic profiling of plasma reveals altered amino acid levels in asthma. (**A**) Heatmap of hierarchical clustering analysis of metabolite levels associated with organic acids for the control group (*n* = 8) and asthma group (*n* = 8) from the screening cohort. Hierarchical clustering analysis. (**B**) Bubble plot of enriched metabolites in KEGG pathways. (**C**) Metabolites associated with the proline metabolism pathway in the control group (*n* = 8) and asthma group (*n* = 8) from the screening cohort. Unpaired, 2-tailed Mann-Whitney test. (**D**) Plasma level of proline in the control group (*n* = 30) and asthma group (*n* = 34) from the validation cohort. Unpaired, 2-tailed Student’s *t* test. (**E**) Plasma level of PYCR1 in the control group (*n* = 30) and asthma group (*n* = 34) from the validation cohort. Unpaired, 2-tailed Student’s *t* test. (**F**) ROC curve of proline for asthma diagnosis. (**G**) ROC curve of PYCR1 for asthma diagnosis. (**H**) ROC curve of PYCR1 and proline for asthma diagnosis. Bar graphs and data are presented as the means ± SEMs. *P < 0.05, **P < 0.01, ***P < 0.001, ****P < 0.0001.

To identify different metabolite levels between patients with asthma and healthy controls, we collected plasma from patients in the screening cohort, and the demographic and clinical data are shown in Table 1. We conducted metabolomics profiling of plasma samples by ultrahigh-performance liquid chromatography–high-resolution mass spectrometry (Supplemental Figure 1A; supplemental material available online with this article; https://doi.org/10.1172/jci.insight.167395DS1). Afterward, the orthogonal projections to latent structures-discriminant analysis (OPLS-DA) plots showed a significant difference in metabolic patterns between the asthmatic and healthy groups (Supplemental Figure 1B). Interrelationships among these metabolic categories were displayed in a circular layout, which revealed that organic acids and derivatives were vital components of differential metabolites (Supplemental Figure 1C). Some organic acids and derivatives, such as ornithine and valyl-proline, were elevated or decreased in individuals with asthma compared with the control group (Figure 1A). To further explore the most prominent metabolic pathways, we conducted enrichment and pathway analysis of all metabolites. Upon Kyoto Encyclopedia of Genes and Genomes (KEGG) pathway enrichment analysis and differential abundance (DA) score analysis, we found that the differential metabolites were enriched in the arginine and proline metabolism pathways between the 2 groups (Figure 1B and Supplemental Figure 1D). The arginine and proline metabolic pathways contain several biochemical activities, i.e., the synthesis of proline. Metabolites included in the process, such as proline, ornithine, and 4-hydroxyproline, showed an increasing tendency in patients with asthma compared with controls (Figure 1C).

Considering that proline metabolism links ornithine and produces 4-hydroxyproline, which was elevated in patients with asthma, it was necessary to measure proline in a larger cohort to verify the alteration. We collected plasma from patients in the validation cohort, and the demographic and clinical data are shown in Table 2. Subsequently, we found an increased level of proline in patients with asthma compared with healthy controls in the verified cohort (Figure 1D). During the process of proline biosynthesis, PYCR1 plays a vital role in catalyzing proline synthesis in the last step (Supplemental Figure 1E). In line with the increased proline, PYCR1 was significantly elevated in patients with asthma compared with the control group (Figure 1E). Moreover, receiver operating characteristic (ROC) curve analysis was performed to evaluate the potential of proline and PYCR1 as biomarkers for asthma. The AUCs of proline, PYCR1, and the combination of proline with PYCR1 to diagnose asthma were 0.951 (95% CI 0.90–1, *P* < 0.05), 0.752 (95% CI 0.64–0.86, *P* < 0.05), and 0.964 (95% CI 0.93–1, *P* < 0.05), respectively, indicating a satisfying ability of proline and/or PYCR1 as biomarkers to distinguish patients with asthma from healthy controls (Figure 1, F–H). Collectively, proline and PYCR1 in plasma from patients with asthma were significantly increased, so they may be involved in asthma pathogenesis.

### PYCR1 gene expression is upregulated in bronchial epithelial cells in asthma.

**Figure 2:**
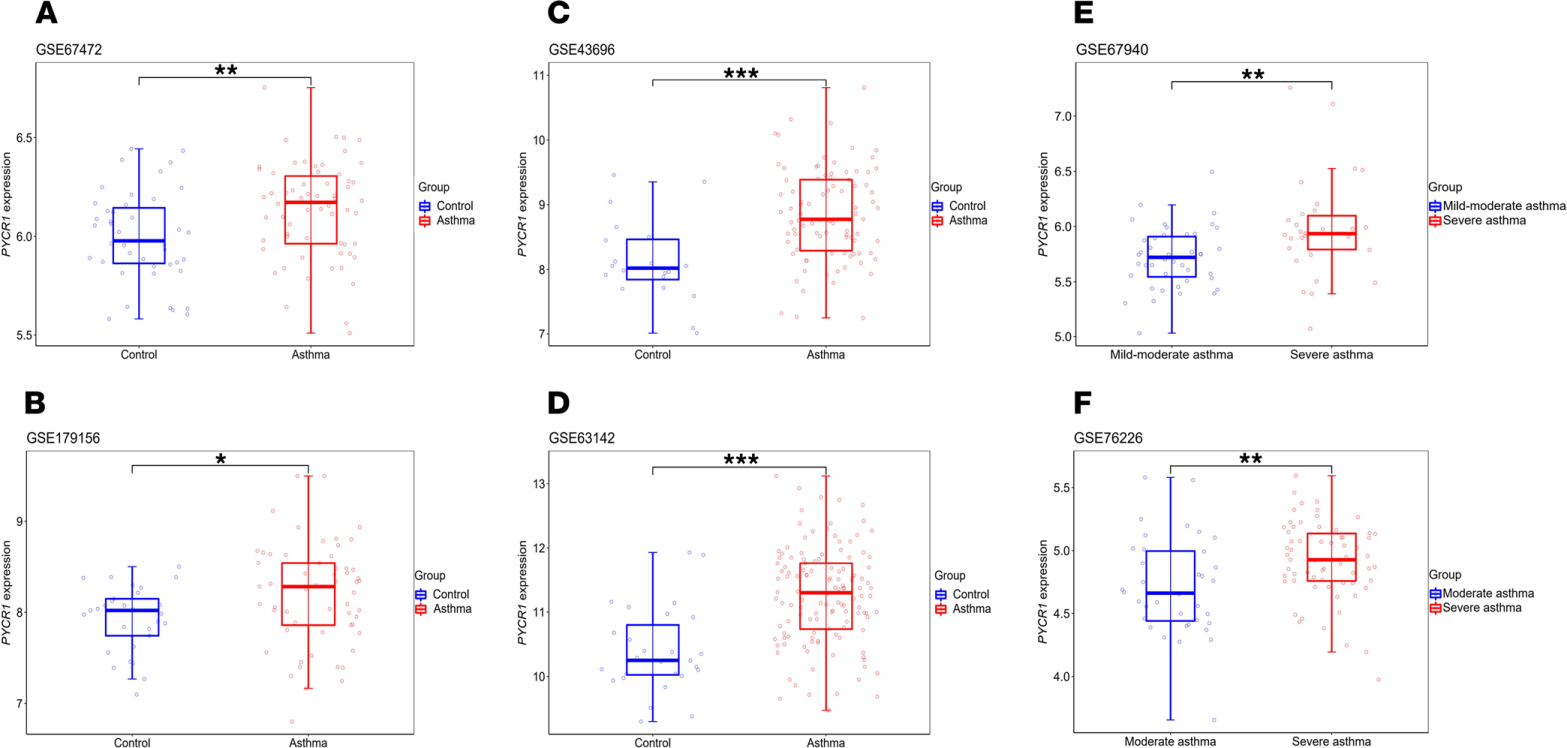
*PYCR1* gene expression is increased in the airway epithelium of patients with asthma compared with healthy controls. (**A**) Box plots of *PYCR1* gene expression in airway epithelial cells from individuals in GSE67472 (*n* control = 43 and *n* asthma = 62). (**B**) Box plots of *PYCR1* gene expression in airway epithelial cells from individuals in GSE179156 (*n* control = 29, *n* asthma = 57). (**C**) Box plots of *PYCR1* gene expression in airway epithelial cells from individuals in GSE43696 (*n* control = 20, *n* asthma = 88). (**D**) Box plots of *PYCR1* gene expression in airway epithelial cells from individuals in GSE63142 (*n* control = 27, *n* asthma = 128). (**E**) Box plots of *PYCR1* gene expression in airway epithelial cells from individuals in GSE67940 (*n* mild-moderate = 43, *n* severe = 30). (**F**) Box plots of *PYCR1* gene expression in airway epithelial cells from individuals in GSE76226 (*n* moderate = 36, *n* severe = 63). Bar graphs and data are presented as medians ± IQRs. Results are shown as box-and-whisker plots. (The middle horizontal line within the box represents the median, boxes extend from the 25th to the 75th percentiles, and the whiskers represent 95% CIs.) Statistical differences were determined with an unpaired, 2-tailed Wilcoxon rank-sum test. *P < 0.05, **P < 0.01, ***P < 0.001.

Since epithelial cells initialize EMT, we next assessed *PYCR1* mRNA expression in the airway epithelium between patients with asthma and healthy controls. The *PYCR1* gene expression profile was analyzed using the Gene Expression Omnibus (GEO) database 2R website. The detailed samples and groups are listed in Table 3. In the GEO GSE67472, GSE179156, GSE43696, and GSE63142 data sets, individuals were divided into a mild-to-moderate or severe asthma group and a healthy control group (30–33). These data sets indicated that *PYCR1* mRNA expression was upregulated in patients with asthma compared with healthy controls (Figure 2, A–D). We further explored the relationship between *PYCR1* mRNA expression and the severity of asthma from GSE67940 and GSE76226 (34, 35), revealing that *PYCR1* mRNA expression was significantly increased in severe asthma compared with mild-to-moderate asthma (Figure 2, E and F). Overall, *PYCR1* mRNA expression was upregulated in the epithelial cells of patients with asthma and correlated with the severity of the disease, which may be associated with epithelial cell status changes.

### Lung PYCR1 overexpression coincides with persistent airway remodeling.

**Figure 3:**
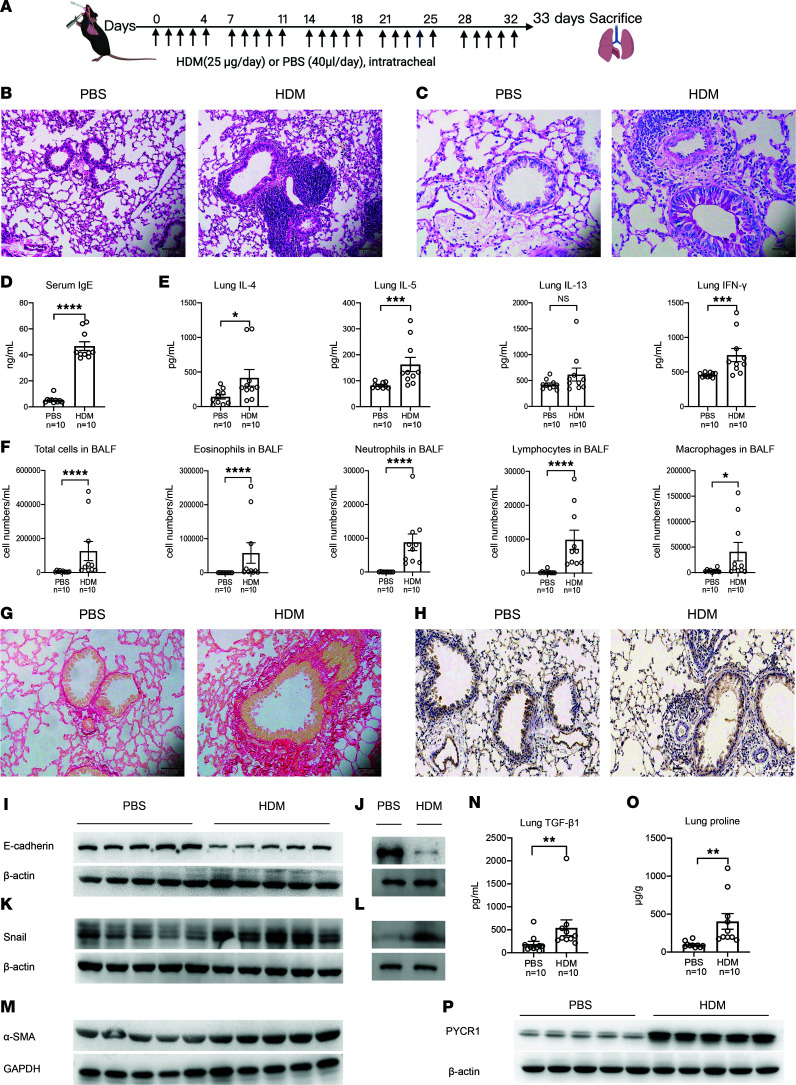
PYCR1 plays a vital role in a mouse model of asthma. (**A**) Experimental design of the chronic asthma model. (**B**) Representative images of lung sections stained with H&E (*n* = 6/group); original magnification 200×; scale bar, 100 μm. (**C**) Representative images of lung sections stained with periodic acid–Schiff (PAS) (*n* = 6/group); original magnification 400×; scale bar, 50 μm. (**D**) The concentration of serum total IgE was measured with ELISA (*n* = 10/group). (**E**) The concentrations of different cytokines (IL-4, IL-5, IL-13, and IFN-γ) in the lung homogenates were measured with ELISA (*n* = 10/group). (**F**) Total cells and differential cell counts in bronchoalveolar lavage fluid (BALF) were measured using a hemocytometer (*n* = 10/group). (**G**) Representative images of lung sections stained with Sirius red (*n* = 6/group); original magnification 400×; scale bar, 50 μm. (**H**) Representative images of immunohistochemical staining for E-cadherin in lung sections (*n* = 3/group); original magnification 400×; scale bar, 50 μm. (**I**) Western blot analysis of E-cadherin expression in lung tissues (*n* = 10/group). (**J**) Western blot analysis of E-cadherin expression in the tracheal epithelium from mice chronically exposed to PBS or HDMs (*n* = 3/group). (**K**) Western blot analysis of Snail expression in lung tissues (*n* = 10/group). (**L**) Western blot analysis of Snail expression in the tracheal epithelium (*n* = 3/group). (**M**) Western blot analysis of α-SMA expression in lung tissues (*n* = 5/group). (**N**) The concentration of TGF-β1 in the lung homogenates was measured with ELISA (*n* = 10/group). (**O**) The proline level of lung tissues was measured using a colorimetric method (*n* = 10/group). (**P**) Western blot analysis of PYCR1 expression in lung tissues (*n* = 10/group). Bar graphs and data are presented as the means ± SEMs. Statistical analysis was performed using unpaired, 2-tailed Student’s *t* test. *P < 0.05, **P < 0.01, ***P < 0.001, ****P < 0.0001.

To explore the alterations in proline and PYCR1 and the underlying mechanisms in asthma, we developed a murine model of chronic asthma (Figure 3A). Compared with mice exposed to PBS, mice exposed to HDM for 5 weeks showed severe lung inflammation and airway remodeling. Hematoxylin and eosin (H&E) and periodic acid–Schiff (PAS) staining demonstrated more inflammation centers and increased goblet cell metaplasia (Figure 3, B and C). Serum IgE was elevated in HDM-challenged mice (Figure 3D). Inflammatory cytokines in lung homogenate, such as IL-4, IL-5, and IFN-γ, were significantly increased in the chronic asthma model (Figure 3E). Meanwhile, immune cellular profiles such as eosinophils were dominant in bronchoalveolar lavage fluid (BALF), which was consistent with previous reports (Figure 3F). In addition, subepithelial collagen deposition surrounding airways was aggravated in HDM-challenged mice (Figure 3G). Notably, the expression of E-cadherin, a marker of the epithelium, was decreased in airway epithelial cells in HDM-challenged mice by immunohistochemistry (Figure 3H). Meanwhile, the expression of E-cadherin was decreased in lung homogenate and mouse tracheal epithelium in HDM-challenged mice (Figure 3, I and J). In contrast, mesenchymal markers, such as Snail, were upregulated in lung tissues and in the mouse tracheal epithelium of chronic asthma (Figure 3, K and L), similar to the increased α-SMA and TGF-β1 in lung homogenate (Figure 3, M and N). Of note, the proline level in lung tissue was higher in the asthma model than in the controls (Figure 3O). Consistently, PYCR1 expression in the lung was upregulated in chronic HDM-challenged mice and was almost twice as high as that in controls (Figure 3P).

In summary, chronic exposure to HDMs revealed an apparent inflammatory pattern and EMT process. Notably, the increase in proline levels and PYCR1 expression in the lung observed with chronic HDM exposure paralleled the occurrence of inflammation and the establishment of lung tissue remodeling.

### HDM stimulation of bronchoalveolar epithelial cells in Pycr1-KO mice restrains EMT.

**Figure 4:**
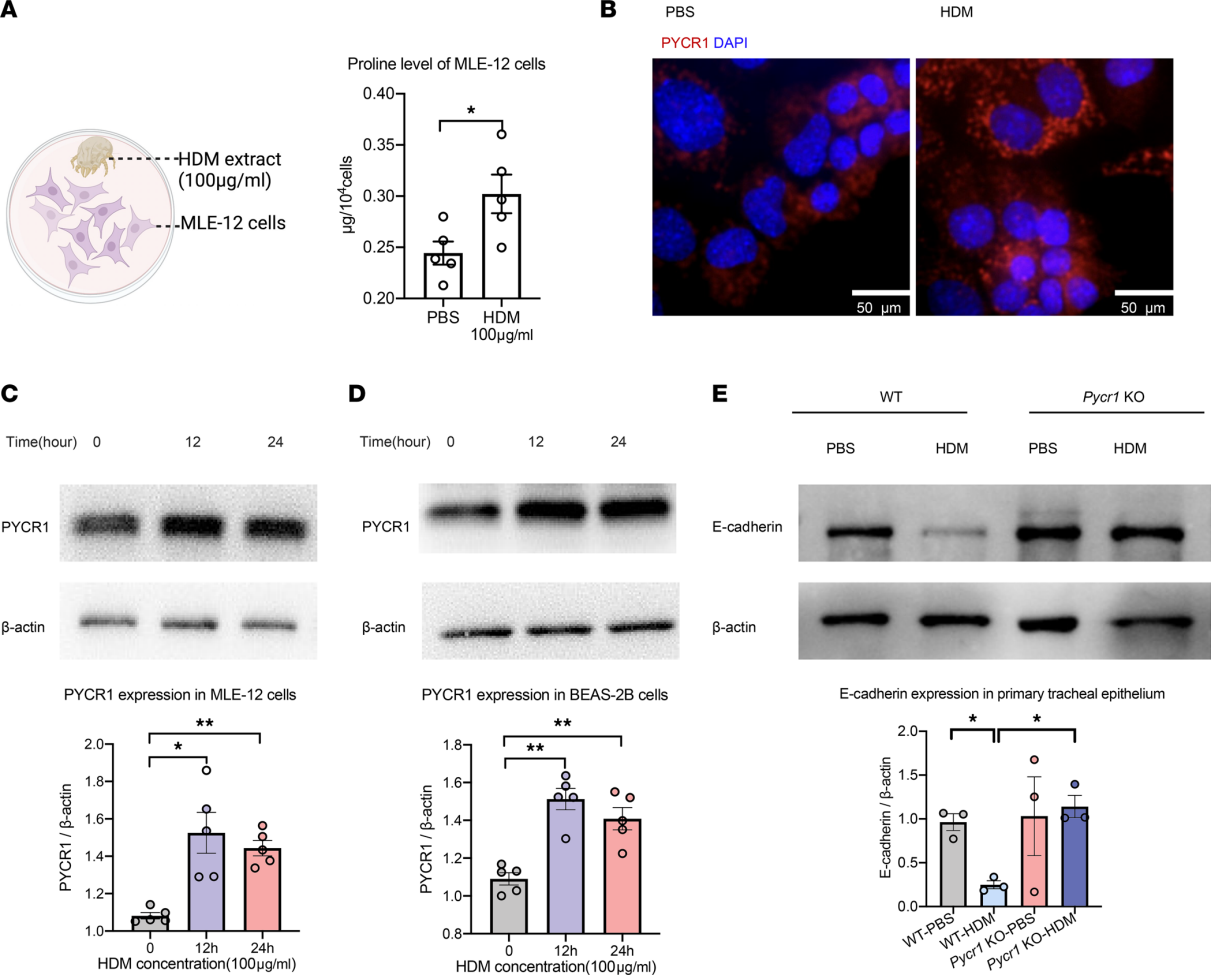
HDM extract promotes proline elevation and PYCR1 expression in bronchial alveolar epithelial cells. (**A**) The level of proline in HDM-stimulated MLE-12 cells for 24 hours was measured by the colorimetric method. (**B**) The level of PYCR1 in MLE-12 cells was identified as costained with DAPI (blue) and PYCR1 (red) and observed by microscopy; original magnification 400×; scale bar, 50 μm. (**C**) Western blot analysis of PYCR1 expression in MLE-12 cells treated with HDM extract (100 μg/mL). (**D**) Western blot analysis of PYCR1 expression in BEAS-2B cells treated with HDM extract (100 μg/mL). (**E**) Western blot analysis of E-cadherin expression in primary tracheal epithelial cells treated with HDM extract from WT mice and *Pycr1*-KO mice (100 μg/mL) for 24 hours. Bar graphs and data are presented as the means ± SEMs. Group comparisons were made using 2-tailed Student’s *t* test (**A**) and paired 1-way ANOVA (**C**–**E**) followed by Bonferroni’s post hoc test. The results are from at least 3 independent experiments. *P < 0.05, **P < 0.01.

Aeroallergens encounter the airway epithelium primarily, activating epithelial cell state changes and EMT processes, which lead to airway remodeling. To explore the roles of proline and PYCR1 in EMT, we treated mouse airway epithelial cells (MLE-12) with HDM extract for 24 hours. Upon HDM stimulation, proline levels were significantly higher (Figure 4A). PYCR1 is essential in proline synthesis. As expected, HDM significantly enhanced the synthesis enzyme of PYCR1 after 24 hours of HDM stimulation (Figure 4, B and C), which was further validated in human bronchial epithelial cells (BEAS-2B, Figure 4D). To determine whether PYCR1 in epithelial cells was required for EMT, we isolated primary mouse tracheal epithelial cells of *Pycr1-*KO and WT control mice at 6–8 weeks of age, which were proved to be of high purity by immunofluorescence of pankeratin (Supplemental Figure 2). We then treated both cell lines with or without HDM for 24 hours. Our results demonstrated that the epithelial marker E-cadherin was decreased in HDM-stimulated primary tracheal epithelial cells in WT mice; however, HDM stimulation in *Pycr1*-deficient mice partially ameliorated the reduction in E-cadherin (Figure 4E). Collectively, proline and *Pycr1* could be involved in EMT-mediated airway remodeling in asthma.

### Knockout of Pycr1 attenuated airway remodeling in an HDM-induced chronic asthma model.

**Figure 5:**
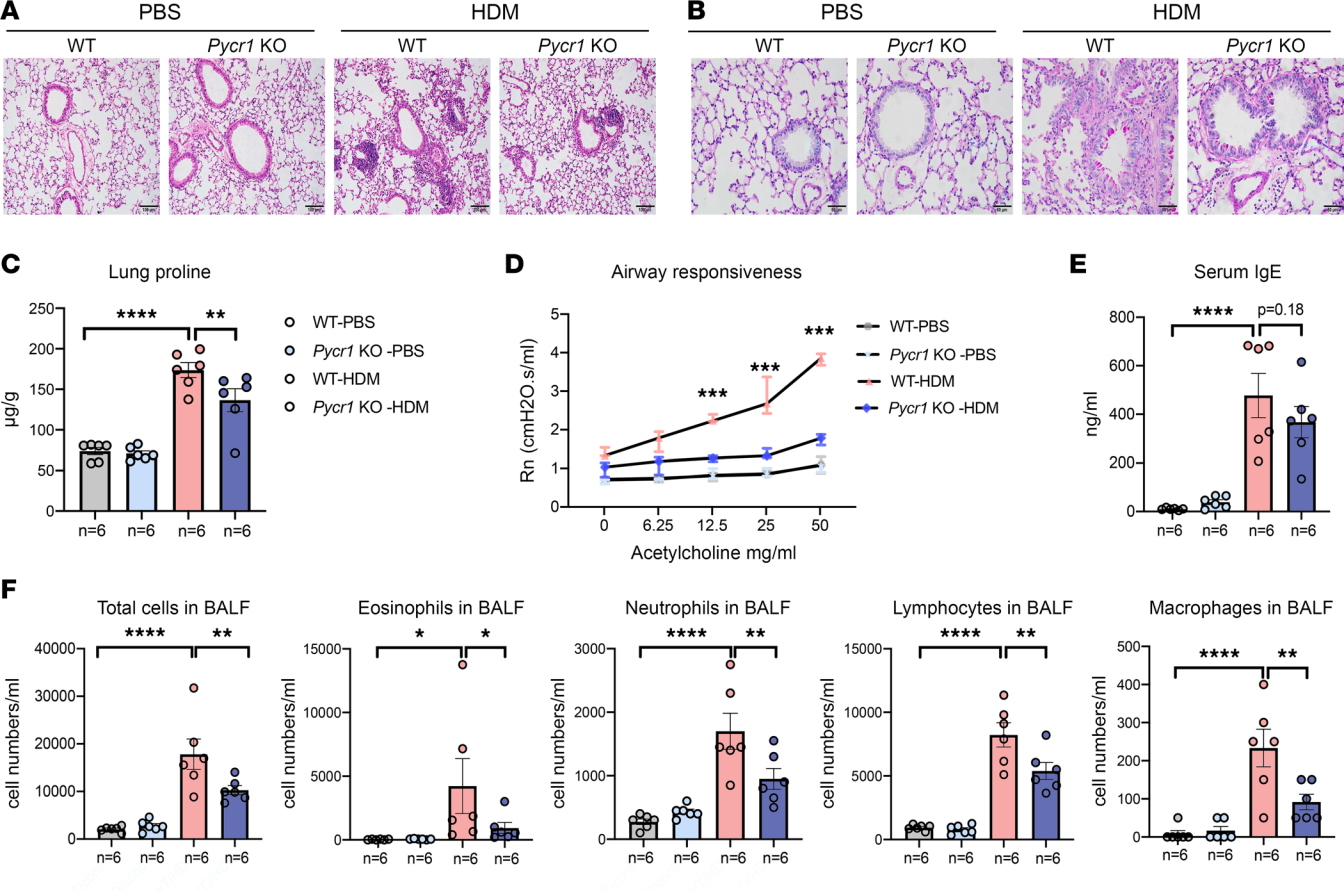
The absence of PYCR1 attenuates the inflammation of HDM-driven asthma. (**A**) Representative images of lung sections stained with H&E (*n* = 6/group); original magnification 200×; scale bar, 100 μm. (**B**) Representative images of lung sections stained with periodic acid–Schiff (PAS) (*n* = 6/group); original magnification 400×; scale bar, 50 μm. (**C**) The level of proline in lung tissues was measured by the colorimetric method among the 4 groups. (**D**) Airway resistance (Rn) values were measured in response to increasing doses of inhaled acetylcholine (0–50 mg/mL) (*n* = 3/group). (**E**) The concentration of serum total IgE was measured with ELISA. (**F**) Total cells and differential cell counts in BALF were measured using a hemocytometer. Statistical analysis was performed using 1-way ANOVA (**C**, **E**, and **F**) followed by Bonferroni’s post hoc test. Group comparisons of airway Rn values were determined with 2-way ANOVA followed by Bonferroni’s post hoc test (**D**). *P < 0.05, **P < 0.01, ***P < 0.001, ****P < 0.0001.

**Figure 6:**
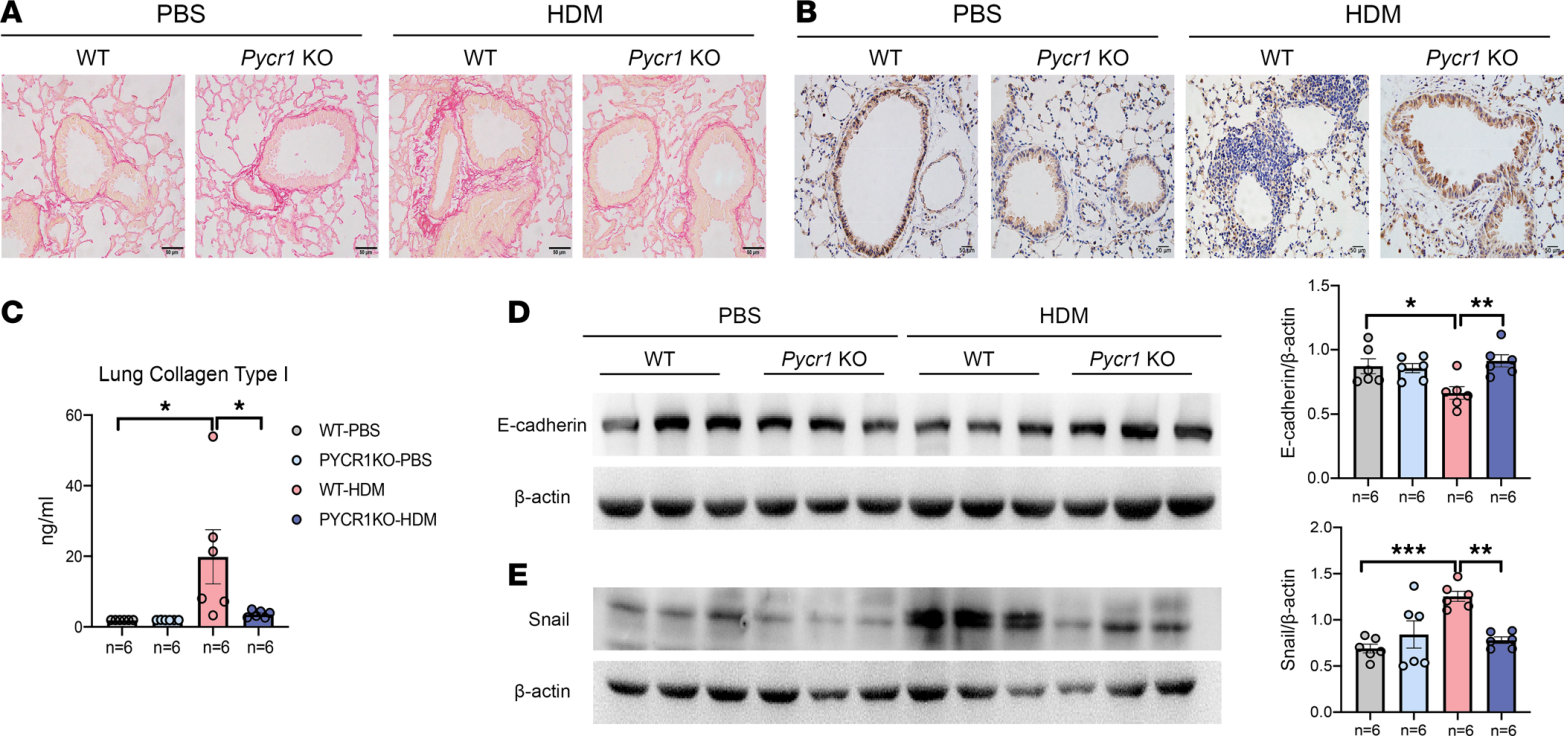
The absence of PYCR1 attenuates the airway remodeling of HDM-driven asthma. (**A**) Representative images of lung sections stained with Sirius red (*n* = 6/group); original magnification 400×; scale bar, 50 μm. (**B**) Representative images of immunohistochemical staining for E-cadherin in lung sections (*n* = 3/group); original magnification 400×; scale bar, 50 μm. (**C**) The concentration of collagen I in the lung homogenates was measured with ELISA. (**D**) Western blot analysis of E-cadherin expression in lung tissues (*n* = 6/group). (**E**) Western blot analysis of Snail expression in lung tissues (*n* = 6/group). Bar graphs and data are presented as the means ± SEMs. Statistical analysis was performed using 1-way ANOVA (**C**–**E**) followed by Bonferroni’s post hoc test. *P < 0.05, **P < 0.01, ***P < 0.001.

Previous studies mentioned that the knockdown of *Pycr1* expression in oral epidermoid carcinoma meng-1 (OECM-1) cells decreased the levels of EMT markers (36). To further elucidate the roles of *Pycr1* in airway remodeling in asthma, we generated *Pycr1* gene–deficient mice by using the CRISPR/Cas9 method. Then, WT mice and *Pycr1-*KO mice were subjected to PBS or HDM to induce the experimental asthma model. In WT mice, the level of inflammation was aggravated in the HDM-driven asthma model compared with that in the PBS-treated model. Conversely, the degree of inflammation was lower in the asthma model of *Pycr1-*KO mice than in WT mice (Figure 5A). PAS staining revealed that the asthma model in *Pycr1-*KO mice exhibited a decreased tendency of mucus secretion compared with that in WT mice (Figure 5B). We also assessed whether *Pycr1* affects proline metabolism in the context of a chronic asthma model, demonstrating that *Pycr1* deficiency reduced the level of proline in the lungs of HDM-challenged mice (Figure 5C). Additionally, we found that *Pycr1* deficiency reduced airway hyperresponsiveness compared with WT mice in the chronic asthma model (Figure 5D). Moreover, total serum IgE and inflammatory cellular profiles in the BALF were significantly diminished in *Pycr1-*KO asthmatic mice (Figure 5, E and F). In mammals, it has been conclusively clarified that PYCR1 regulates proline synthesis to deposit collagen. Consistently, we observed that loss of *Pycr1* mitigated subepithelial collagen deposition around airways, rescued the expression of E-cadherin in airway epithelial cells, and reduced the production of collagen type I in the lung tissue of the asthma model (Figure 6, A–C). Additionally, the expression of E-cadherin was augmented in response to *Pycr1* deficiency, while mesenchymal markers, such as Snail, were downregulated compared with those in WT asthmatic mice (Figure 6, D and E).

Collectively, these results suggested that *Pycr1* was involved in the regulation of inflammation and EMT-associated airway remodeling in the HDM-driven asthma model.

### Pycr1 regulates EMT through the modulation of mitochondrial fragmentation, cellular metabolism, and cell signaling.

**Figure 7:**
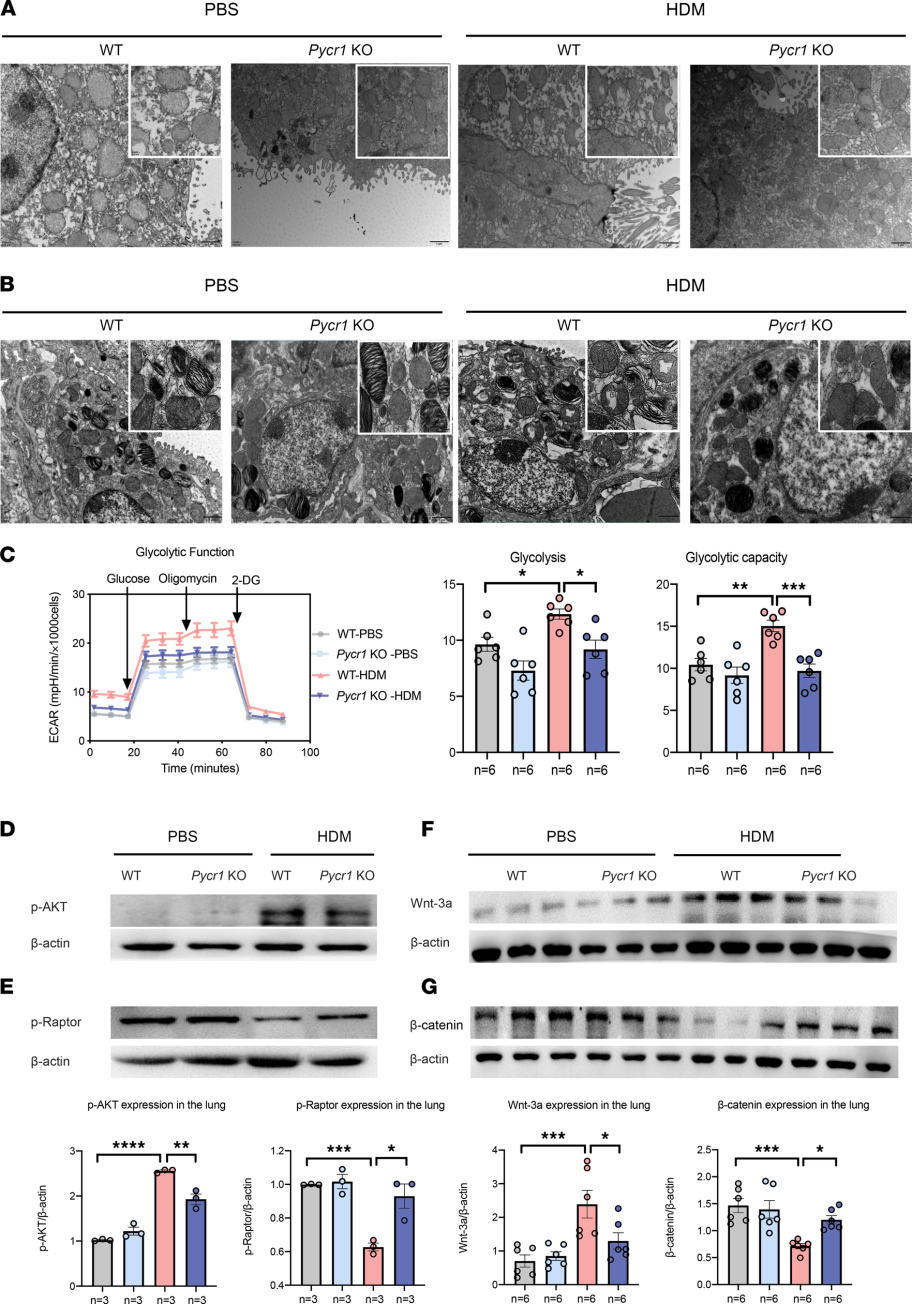
PYCR1 deficiency regulates the mitochondrial structure and cellular metabolism of the airway epithelium and AKT/mTORC1 and Wnt3a/β-catenin signaling ex vivo. (**A**) The mitochondrial morphology alterations in primary tracheal epithelial cells from the 4 groups were observed by transmission electron microscopy (*n* = 3/group); original magnification 12,000×; scale bar, 1 μm. (**B**) The mitochondrial morphology alterations in alveolar epithelial cells from the 4 groups were observed by transmission electron microscopy; original magnification 12,000×; scale bar, 1 μm. (**C**) Representative ECAR profiles, changes in basal ECAR, and glycolytic capacity in primary airway epithelial cells from 4 groups. (**D**) Western blot analysis of p-AKT expression in lung tissues (*n* = 3/group). (**E**) Western blot analysis of p-Raptor expression in lung tissues (*n* = 3/group). (**F**) Western blot analysis of Wnt3a expression in lung tissues (*n* = 6/group). (**G**) Western blot analysis of β-catenin expression in lung tissues (*n* = 6/group). Bar graphs and data are presented as the means ± SEMs. Statistical analysis was performed using 1-way ANOVA followed by Bonferroni’s post hoc test. *P < 0.05, **P < 0.01, ***P < 0.001, ****P < 0.0001.

PYCR1 was dominantly located in mitochondria and played an important role in metabolism; hence, it is natural to investigate whether mitochondrial changes or cellular metabolic process alterations existed in the HDM-driven asthma model. Subsequently, we observed that compared with PBS-treated mice, HDM-treated mice exhibited increased numbers of abnormal mitochondria with swelling, vacuolation, and depletion of cristae in primary tracheal epithelial cells beneath cilia cells. After continuous HDM exposure, mitochondrial morphology defects were partially attenuated in *Pycr1-*KO mice compared with WT mice, as judged by less damage to mitochondrial vacuolation and the structure of cristae in primary tracheal epithelial cells (Figure 7A). The results were consistent with the change in mitochondrial morphology in type II alveolar epithelial cells (AEC2s) marked by lamellar bodies (Figure 7B), revealing that *Pycr1* deficiency played a protective role against mitochondrial damage after exposure to HDMs. For cellular metabolism, the extracellular acidification rate (ECAR) was determined to quantify the glucose metabolism balance. Basal and peak glycolysis were upregulated in AEC2s from WT asthmatic mice, resulting in increased glucose consumption and lactate production. In contrast, glycolysis and glycolytic capacity were decreased in AEC2s from *Pycr1-*KO asthmatic mice (Figure 7C). These observations indicated that *Pycr1* deficiency relieved EMT by reducing mitochondrial disruption and modulating cellular metabolism.

Thus far, the transition from epithelial to mesenchymal state relative to mitochondria relies on stimulatory cues, such as the WNT, TGF-β, AKT, and mTOR signaling pathways (37, 38). To further dissect how *Pycr1* regulates airway remodeling, we examined the expression of WNT3a/β-catenin and AKT/mTORC1 in lung tissue. In line with a switch from the epithelial to mesenchymal state, the expression levels of WNT3a and phosphorylated (p-) AKT were upregulated, while the expression levels of β-catenin and p-Raptor were downregulated in WT asthmatic mice compared with WT controls. The expression of these signaling proteins was conversely altered in *Pycr1-*KO asthmatic mice compared with WT asthmatic mice, indicating that epithelial cells remained in an epithelial state in *Pycr1-*KO asthmatic mice (Figure 7, D–G). Together, these results revealed that *Pycr1* deficiency may inhibit the WNT and AKT signaling pathways, which subsequently prevents progression to severe airway remodeling, even in the context of successive exposure to allergens.

### Inhibition of endogenous proline or deprivation of exogenous proline partially ameliorates airway remodeling in a murine chronic asthma model.

**Figure 8:**
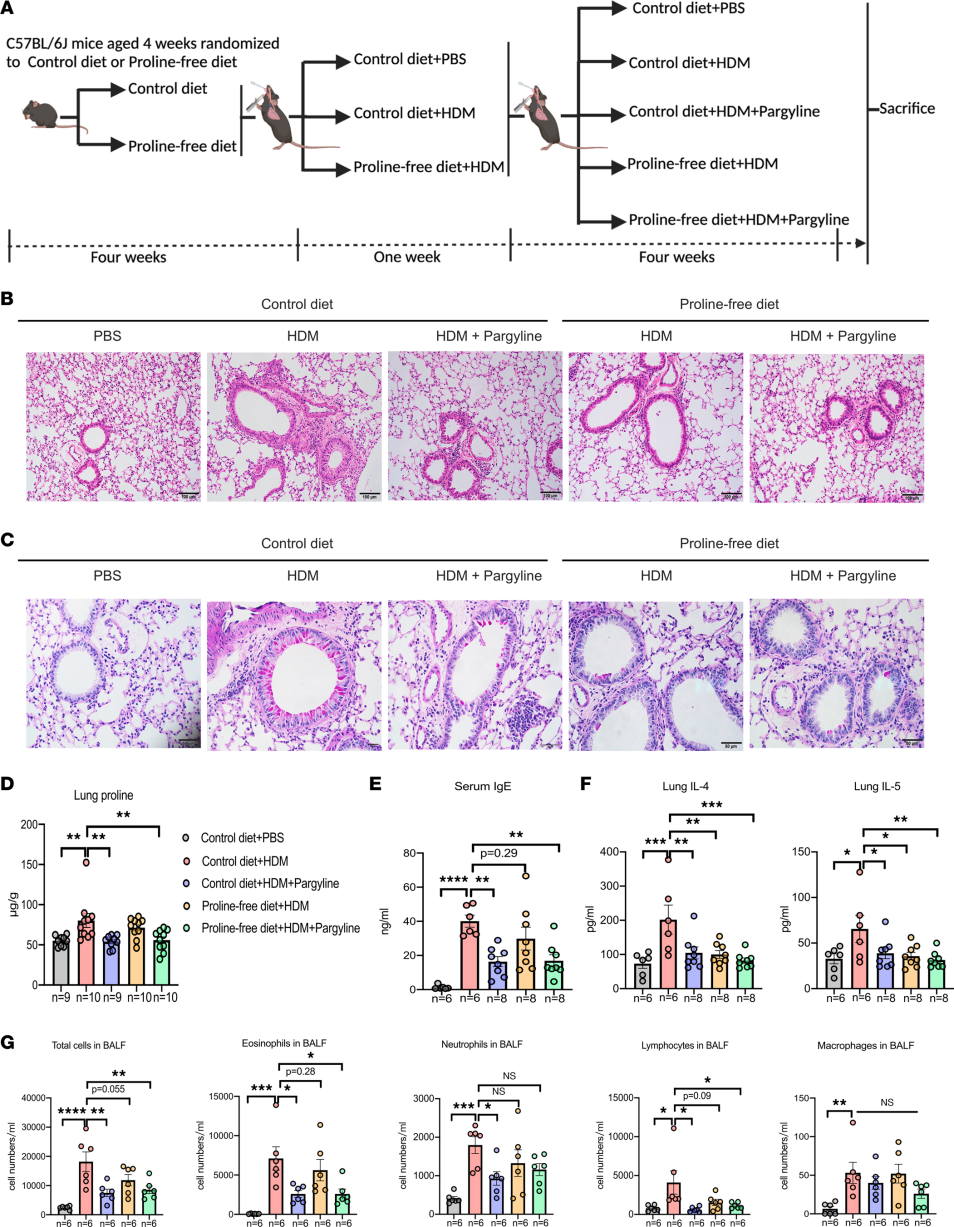
Inhibition of PYCR1 or starvation with exogenous proline partially ameliorated airway inflammation in a murine chronic asthma model. (**A**) Schematic diagram of intervention with pargyline and a PFD in the HDM-induced chronic asthma model protocol. (**B**) Representative images of lung sections stained with H&E (*n* = 6/group); original magnification 200×; scale bar, 100 μm. (**C**) Representative images of lung sections stained with periodic acid–Schiff (PAS) (*n* = 6/group); original magnification 400×; scale bar, 50 μm. (**D**) The level of proline in lung tissues was measured by the colorimetric method among the 5 groups (*n* = 9–10/group). (**E**) The concentration of serum total IgE was measured with ELISA (*n* = 6–8/group). (**F**) The concentrations of IL-4 and IL-5 in the lung homogenates were measured with ELISA (*n* = 6–8/group). (**G**) Total cells and differential cell counts in bronchoalveolar lavage fluid were measured using a hemocytometer (*n* = 6/group). Bar graphs and data are presented as the means ± SEMs. Statistical analysis was performed using 2-way ANOVA followed by Bonferroni’s post hoc test. *P < 0.05, **P < 0.01, ***P < 0.001, ****P < 0.0001.

Sources of proline in mammals include endogenous production and exogenous ingestion. To investigate the effect of proline metabolism on HDM-induced airway remodeling, we adopted 3 methods in a murine asthma model, including inhibition of PYCR1, a proline-free diet (PFD), and combined therapy (Figure 8A).

**Figure 9:**
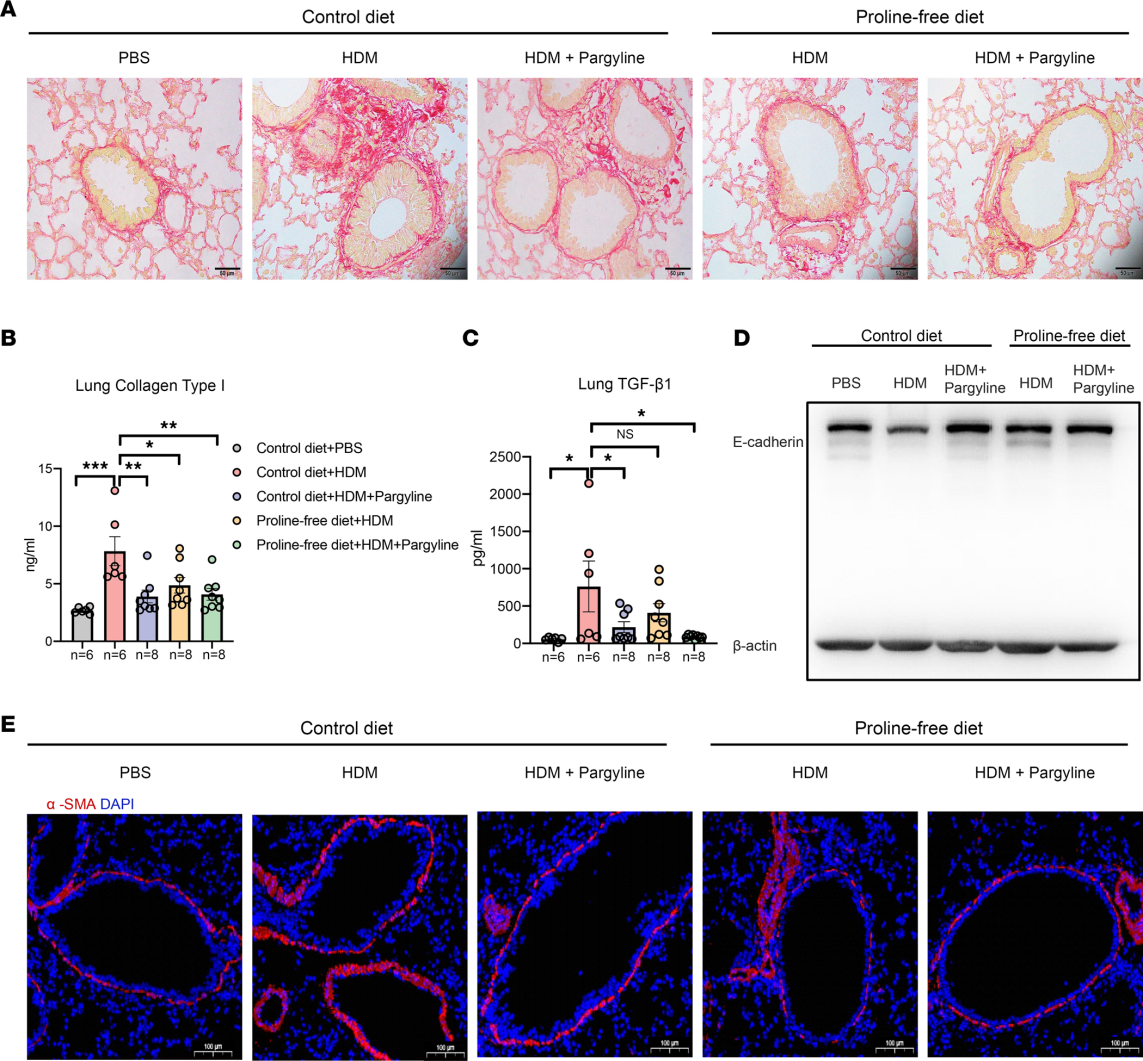
Inhibition of PYCR1 or starvation with exogenous proline partially ameliorated airway remodeling in a murine chronic asthma model. (**A**) Representative images of lung sections stained with Sirius red (*n* = 6/group); original magnification 400×; scale bar, 50 μm. (**B**) The concentration of collagen I in the lung homogenates was measured with ELISA (*n* = 6–8/group). (**C**) The concentration of TGF-β1 in the lung homogenates was measured with ELISA (*n* = 6–8/group). (**D**) Western blot analysis of E-cadherin expression in lung tissues (*n* = 3/group). (**E**) The expression of α-SMA around airways was identified as costained with DAPI (blue) and α-SMA (red) and observed by microscopy (*n* = 3/group); original magnification 200×; scale bar, 100 μm. Bar graphs and data are presented as the means ± SEMs. Statistical analysis was performed using 2-way ANOVA followed by Bonferroni’s post hoc test. *P < 0.05, **P < 0.01, ***P < 0.001.

According to these strategies, we first evaluated whether the inhibition of endogenous proline synthesis via the inhibition of PYCR1 could block EMT-associated airway remodeling in a chronic asthma model. To this end, we established the control diet + HDM + pargyline model by intraperitoneal injection of 100 mg/kg pargyline, a small molecule reported to inhibit the activity of PYCR1, from the start of the HDM challenge phase. In the chronic asthma model, HDM challenge led to increased lung inflammation, BALF cellular profiles, collagen deposition, concomitantly decreased expression of E-cadherin, and enhanced expression of α-SMA. Notably, compared with HDM-induced asthmatic mice, inflammatory sections and mucus secretions of lung tissue were decreased by inhibiting PYCR1 (Figure 8, B and C). In addition, inhibition of PYCR1 reduced the proline level in lung tissue compared with that in HDM-driven asthmatic mice (Figure 8D). Meanwhile, serum IgE shared a similar tendency with proline alteration between the 2 groups (Figure 8E). Furthermore, inhibition of PYCR1 had a significant effect on reducing HDM-induced production of IL-4 and IL-5 in lung homogenates (Figure 8F). As expected, inhibition of PYCR1 significantly reduced the differential inflammatory cell counts (Figure 8G). Moreover, asthma is characterized by airway remodeling with collagen deposition and fibrosis. Regarding remodeling, the area of collagen deposition around airways and the production of collagen I in lung tissues were decreased after inhibiting PYCR1 compared with HDM-driven asthmatic mice (Figure 9, A and B). In addition, mice in the pargyline group exhibited a significant reduction in TGF-β1 expression in lung tissue (Figure 9C). Concerning EMT-related markers, E-cadherin expression in the lung was significantly elevated in pargyline-treated mice compared with HDM-challenged mice (Figure 9D). Moreover, HDM challenge enhanced the fluorescence intensity of α-SMA, which implies thickening of the small airways. Of note, in comparison with HDM-induced asthmatic mice, mice treated with pargyline experienced a deterioration of the intensity, indicating the amelioration of airway remodeling (Figure 9E).

To further assess the preventative effect of exogenous proline deprivation, mice were fed a PFD. Compared with HDM-induced allergic mice fed a control diet, HDM-induced allergic mice fed PFD had significantly reduced inflammatory levels, which can be partially inferred from some markers, e.g., inflammatory sections, mucus secretion, and inflammatory cytokines (Figure 8, B, C, and F). Additionally, PFD mitigated airway remodeling due to a decreased area of subepithelial collagen deposition around airways, reduced production of collagen I in lung tissue, and restrained EMT (Figure 9, A–E).

To explore the possible synergistic effect, we combined pargyline and PFD in an asthma model. We found that the combined therapy revealed similar effects on inflammation and remodeling as pargyline or PFD. However, combined therapy did not exhibit significant improvement when compared with pargyline or PFD alone.

Together, these results demonstrated that restriction of endogenous proline by inhibition of PYCR1 and reduction of exogenous proline via a PFD, to some extent, attenuate inflammation and airway remodeling in a murine asthma model induced by chronic HDM exposure.

## Discussion

Our study described a plasma metabolomic signature related to asthma. Amino acids and related enzymes in the arginine and proline pathways, especially proline and PYCR1, were distinctively altered in patients with asthma compared with controls. In the HDM-induced asthma model characterized by airway inflammation and remodeling, proline and PYCR1 were increased. *Pycr1* deficiency restrained inflammation, airway hyperresponsiveness (AHR), and EMT, which may be associated with the regulation of mitochondrial damage, cellular metabolomic imbalance, and WNT3a/β-catenin and AKT/mTORC1 signaling activation. In addition, we found that a reduction in endogenous or exogenous proline may attenuate inflammation and remodeling in asthma, demonstrating that *Pycr1* deficiency or starvation of proline could prevent airway remodeling in HDM-induced allergic asthma, warranting further investigation of proline and PYCR1 as therapeutic targets for asthma.

Previous studies have identified several circulating metabolites in asthma that differ from those in healthy individuals via serum or plasma-based metabolic profiling through nuclear magnetic resonance high-performance and liquid chromatography-tandem mass spectrometry methods (39, 40). Several studies have mentioned that the plasma concentrations of lipids and amino acids, including sphingolipids, uric acid, tryptophan, and arginine or proline, correlate with the prevalence of asthma (41, 42). Abdulnaby et al. revealed higher uric acid in patients with asthma (43), while several documents reported alterations in metabolites of the arginine metabolism pathway in the asthma group (41, 44, 45). Moreover, Comhair et al. reported elevated levels of taurine and bile acids in asthmatic plasma (46). Reinke et al. conducted serum-based metabolomics via liquid chromatography–high-resolution mass spectrometry and found significant differences in lipid metabolism in patients with asthma (47). In our study, the healthy controls and asthma groups were characterized by several differentially abundant metabolites. Of particular interest were the increases in proline, ornithine, and 4-hydroxyproline, which strongly contributed to the separation of patients with asthma from controls and represented major nodes of metabolite clustering in the arginine and proline metabolism pathways. Meanwhile, *PYCR1* was elevated at the mRNA level in human bronchial epithelial cells from GEO data sets with large populations and increased at the protein level in plasma from our cohort. Therefore, the elevation of proline and *PYCR1* in bronchial epithelial airways in asthma may be involved in the pathogenesis of asthma. However, the relationship between proline/*PYCR1* and the clinical features of asthma remains unsolved.

The HDM-induced allergic model for 5 weeks is commonly used to mimic key features of asthma. Furthermore, PYCR1 is a mitochondrial enzyme that catalyzes the conversion of pyrroline-5-carboxylate to proline, which is involved in invasiveness and inflammation. Kuo et al. overexpressed and knocked down *Pycr1* in OECM-1 cells and revealed that *Pycr1* promotes the expression of N-cadherin in vitro (36). The mRNA transcript level of the *Pycr1* gene was upregulated in lung homogenates of an HDM-induced asthma model for 14 days (29); however, the association of the expression of the PYCR1 protein with airway remodeling remains unclear. In patients with asthma, airway remodeling is related to EMT, which may be attributed to subepithelial deposition of collagen and fibrosis, leading to a reduction in lung function. Similar to previous studies, we observed the presence of EMT characterized by decreased E-cadherin expression and increased α-SMA or Snail expression, along with increased collagen I, which was associated with the severity of airway remodeling in the HDM-induced asthma model (16, 48). Concomitantly, proline levels and PYCR1 expression were elevated, accompanied by EMT after HDM stimulation, while the effect of *Pycr1* deficiency on proline and airway remodeling needs to be explored. The proline level of the homogenous lung was similar between WT mice and *Pycr1-*KO mice after PBS administration, revealing that other sources exist to maintain the balance of proline besides synthesis through PYCR1. However, loss of *Pycr1* reduced proline in the lung after HDM exposure. Conversely, the EMT markers experienced a reversal in asthma in the *Pycr1-*KO model compared with the WT model ex vivo and in vitro, accompanied by fewer inflammatory cells, diminished secretion of mucus, decreased airway hyperresponsiveness, and reduced collagen I. Hence, elevated proline may promote EMT-mediated airway remodeling through an increase in PYCR1 in a chronic asthma model.

Thus far, there are few mechanistic investigations related to metabolites in asthma and potential targets to treat asthma. HDM-induced EMT originates from crosstalk between bronchoalveolar epithelial cells and multiple immune cells. Upon exposure to allergens, epithelial cells act as the first defense through the release of EGF and TGF-β to prompt fibroblasts and the loss of epithelial junctions, which eventually boost airway remodeling (8, 49, 50). In our study, mice lacking *Pycr1* developed relatively mild allergic airway inflammation and remodeling compared with WT mice. For this reason, and because PYCR1 protein is located in mitochondria and *PYCR1* mRNA is higher in bronchial cells from patients with asthma, we hypothesized that PYCR1 might influence the biology of epithelial cells and mitochondria. Our results demonstrated that HDM exposure caused mitochondrial disruption of fission in the tracheal epithelium and AEC2s, which may explain why we detected PYCR1 in human plasma. On the one hand, recent publications reported that they found cell-free mitochondria in the blood (51). On the other hand, mitochondrial fission overwhelmed fusion after allergen stimulation in AEC2s, leading to the release of enzymes into circulation (52–54). More importantly, we showed that airway epithelial cells from HDM-challenged *Pycr1-*KO mice have dramatically fewer mitochondrial disruptions than epithelial cells from HDM-challenged WT mice. Since PYCR1-mediated mitochondrial damage might have downstream influences on metabolism, we further elucidated that airway epithelial cells experienced a metabolic shift from reliance on oxidative phosphorylation to a glycolysis-dependent status in HDM-challenged WT mice. Nevertheless, airway epithelial cells in *Pycr1-*KO mice did not exhibit metabolic reprogramming as shown by a marked decrease in the uptake and use of glucose as a preference for metabolism compared with those in WT mice after HDM exposure. In accordance with earlier studies (55), upregulation of the AKT/mTORC1 signaling pathway, characterized by increased expression of p-AKT and decreased expression of p-Raptor in our study, enhanced glycolysis and synthesis of amino acids. Moreover, the interplay between AKT/mTORC1 and WNT3a/β-catenin signaling may be involved in asthmatic airway remodeling (56). In contrast, *Pycr1* deficiency partially inhibited the activation of AKT/mTORC1 and WNT3a/β-catenin signaling, which may account for the inhibition of EMT after HDM challenge.

Since PYCR1 mediates elevated proline and significant molecular abnormalities of epithelial cells during asthmatic remodeling, it is reasonable to speculate whether inhibition of PYCR1 activity or restriction of exogenous proline could affect airway remodeling in clinical relevance. In terms of inhibitors, Oudaert et al. reported that the combination of pargyline and bortezomib instead of pargyline alone could reduce tumor burden in a murine multiple myeloma model (57). Our results revealed that pargyline alone or in combination with PFD exhibited marked efficacy in the treatment of inflammation and remodeling in asthma. Pargyline is a modest inhibitor of PYCR1, whereas it can inhibit lysine-specific demethylase 1 activity and is clinically used as an antihypertension or antidepression agent (58). Concerning nonspecific inhibition, we cannot exclude side effects due to off-target effects. However, we observed that pargyline alone or in combination with PFD reduced proline levels in homogenous lungs. Regarding exogenous proline, although Sahu et al. revealed that PFD for 1 month could reduce proline levels in murine plasma (59), our study indicated that HDM-challenged mice after 9 weeks of PFD did not exhibit a significant decrease in proline in lung tissue. Concomitantly, PFD failed to reduce some markers, including serum IgE, inflammatory cells in BALF, and TGF-β1, in lung tissue compared with the control diet after 5 weeks of HDM administration. The sources of proline and isozymes of PYCR1 may partially explain the incomplete attenuation of inflammation and remodeling. One source of proline is the prolidase-catalyzed reaction of various proteins, which is difficult to completely block (60). Another supply of proline comes from the conversion of glutamine or ornithine into proline under the catalysis of PYCR2 or PYCRL, which were identified as isoforms of PYCR1 and may be insensitive to pargyline (61). To some extent, PFD showed efficacy in the treatment of inflammation, as inferred from changes in histology and IL-4 and IL-5 levels in lung tissues. Moreover, 9 weeks of PFD led to a reduction in collagen I biosynthesis and reversed the expression of E-cadherin and α-SMA after HDM exposure. Proline comprises approximately 10% of the amino acids of collagen (60), which may at least partially explain why proline starvation alleviated asthmatic remodeling.

In summary, our study demonstrates that proline metabolism, especially PYCR1, plays a vital role in asthma. PYCR1, which is upregulated in human asthmatic epithelial cells and HDM-induced murine allergic asthma models, prompts the synthesis of proline, which is critical for airway inflammation, hyperresponsiveness, and remodeling by modulating mitochondrial damage and metabolic reprogramming in airway epithelial cells, along with the activation of downstream pro-remodeling AKT/mTORC1 and WNT3a/β-catenin signaling. Inhibition of PYCR1 or reduction of proline relieved airway remodeling in asthma. We propose that agents that selectively target PYCR1 activity and block the source of proline would likely be an efficacious therapy for asthma treatment.

## Methods

### Study population and sample collection

Two cohorts, including an identification cohort and a verification cohort, were recruited to investigate the metabolomic features in patients with asthma recruited from the First Affiliated Hospital of Nanjing Medical University. The identification cohort consisted of 8 patients with asthma and 8 healthy controls. According to the 2019 Global Initiative for Asthma guidelines (62), asthma was diagnosed by a respiratory physician based on self-reported symptoms combined with reversible airflow limitation using a spirometer. Certified technicians performed pulmonary function tests before or after bronchodilator inhalation (salbutamol 400 μg) according to a standard protocol. Airflow limitation was defined as a postbronchodilator FEV_1_ to forced vital capacity ratio of less than 0.70. The guidelines defined positive bronchodilator reversibility as an increase in FEV_1_ > 200 mL and >12% from baseline 20 minutes after bronchodilator inhalation. Healthy volunteers with no history of allergic disease or evidence of asthma were used as controls. Blood samples were collected on the day of pulmonary function and were centrifuged to obtain plasma. Plasma samples were stored at –80°C until metabolite profiling. The samples were from initial collections only without any freeze/thaw cycles.

The verification cohort consisted of 34 patients with asthma and 30 healthy controls. Participants were enrolled with inclusion criteria identical to those for the identification cohort, and their plasma samples were used to measure selected metabolites or related enzymes.

### Metabolomic profiling and data processing

Metabolomic profiling was conducted with plasma samples from the identification cohort via ultrahigh-performance liquid chromatography–high-resolution mass spectrometry (Vanquish, Thermo Fisher Scientific; Q Exactive Orbitrap, Thermo Fisher Scientific) according to previously described methods (63, 64). Briefly, frozen plasma samples were processed for instrumental analysis. Meanwhile, part of each sample was used as quality control (QC) samples. The mass spectrometer was operated in positive and negative ion modes. One blank sample and each QC sample were measured to monitor the reproducibility and stability of the instruments. To identify metabolite compounds, accurate mass (*m/z*, ±5 ppm), retention time (±30 seconds), and spectral patterns were analyzed at the Metabolomics Standards Initiative level. Further structural annotations were searched in the public Human Metabolome Database based on multiple chemical details, such as the accurate mass, isotopic patter fit scores, and MS/MS spectra similarity. The identified metabolites were further checked manually to avoid false positives and guarantee accuracy. Finally, data from each sample were normalized or log-transformed before statistical analysis. To observe data patterns, OPLS-DA was conducted with R software for multivariate analysis. To identify different levels of metabolites between asthma and control subjects, other univariate analyses, such as Student’s *t* test, *P* value false discovery rate adjustments, DA score for enrichment, and pathway analysis, were conducted by R software.

### Quantification of selected metabolites

The plasma samples from the verification cohort were used to detect the levels of proline (Solarbio, BC0290) following the manufacturer’s protocols. The plasma level of PYCR1 was measured by ELISA according to standard protocols (LMAI Bio, LM-11556H). To evaluate the difference in the levels of metabolites and related enzymes between groups, Student’s *t* test or the Mann-Whitney *U* test was performed depending on the distribution of data, which were subsequently applied for ROC curve analysis to distinguish patients with asthma and controls.

### Microarray data information and PYCR1 expression analysis

The human gene expression profiles from GSE67472, GSE179156, GSE43696, GSE63142, GSE67940, and GSE76226 were obtained from the GEO database to filter *PYCR1* expression, analyzed by the GEO2R tool, and then plotted with R software. *PYCR1* was screened according to the conditions of *P* < 0.05.

### Animals

*Pycr1-*KO mice on a C57BL/6 background were purchased from Cyagen Biosciences company (KOCMP-06105-*Pycr1*). *Pycr1*-KO mice were generated using CRISPR/Cas9 technology. Briefly, a single guide RNA (sgRNA) was designed to target exons 3–6 of *Pycr1*. *Cas9* mRNA and sgRNA were obtained by in vitro transcription and then microinjected into the cytoplasm of C57BL/6 mouse embryos at the pronuclear stage. The injected embryos were implanted into the oviducts of pseudopregnant mice for the production of *Pycr*1-KO mice. Finally, the mutation in *Pycr1* in the F0 founder mice was identified by sequence analysis and polymerase chain reaction (PCR). Breeding with WT mice was performed for F1 animal generation. For mouse genotyping, genomic DNA was extracted, and then PCR was performed to identify the genotype of the mice. Heterozygous recombinant mice were backcrossed to parent heterozygous mice for 5 generations. WT mice and *Pycr1-*KO mice were housed under specific pathogen–free conditions at Nanjing Medical University and were randomized to the control or other groups. The mouse models used in our study are described below.

### Induction of chronic allergic airway disease

Female C57BL/6 mice (Laboratory Animal Center, Nanjing Medical University, Nanjing, China) and *Pycr1-*KO mice aged 6–8 weeks were subjected to either the control or the asthma model condition. To induce a chronic asthma model, mice were exposed to HDM extract (25 μg of protein in 40 μL saline, Greer Laboratories) intratracheally under isoflurane anesthesia, which lasted for 5 days per week for up to 5 weeks. Control mice received 40 μL sterile PBS intratracheally. Finally, the mice were euthanized 24 hours after the last challenge (day 33) to collect serum, BALF, and lung tissue for further experiments.

### Proline restriction in chronic allergic airway disease

#### PFD in an in vivo HDM model.

A control diet and PFD were purchased from Jiangsu XIETONG Company based on a previous study (59). For allergic disease prevention studies of proline deprivation, 4-week-old female mice were fed a control diet or PFD for 4 weeks prior to HDM administration and maintained on the same diet until the day of sacrifice.

#### Inhibition of PYCR1 in an in vivo HDM model.

Eight-week-old female mice were sensitized to HDM from days 0 to 4. Following sensitization to HDM, animals were randomized to 1 of the 5 groups: HDM + control diet, HDM + control diet + pargyline (MilliporeSigma, P8013), HDM+ PFD, and HDM + PFD + pargyline. From day 7, mice underwent intraperitoneal injection of either 100 mg/kg PYCR1 inhibitor (pargyline) in 200 μL sterile PBS or 200 μL sterile PBS alone for 5 days per week until the end of the study (57).

### Measurement of AHR

Airway responsiveness was measured with ketamine (100 μg/g) and xylazine (10 μg/g) for anesthetization at 24 hours after the final challenge using mechanical ventilation in a FlexiVent apparatus (SCIREQ). The lung Rn of each group was measured in response to increasing doses of aerosolized acetylcholine (0, 6.25, 12.5, 25, 50 mg/mL, MilliporeSigma, A6625-25G) as described previously (65, 66).

### Serum collection

After AHR measurement, murine blood was collected and incubated for 2 hours at room temperature without anticoagulant and then centrifuged at 2,000*g* for 10 minutes to isolate serum. The serum was stored at −20°C for the IgE test.

### BALF and differential cell counts

BALF was obtained by washing the lung 3 times with 0.5 mL of buffer (PBS containing 1% BSA and 1 mmol EDTA) on ice. After centrifugation at 500*g* for 5 minutes at 4°C, the supernatant (cell-free BALF) was discarded. The cell pellets were resuspended in 200 μL PBS, and differential cell counts were subsequently measured using a hemocytometer (ADVIA 2120i, Siemens).

### Lung histology

The lower lobe of each left lung was fixed in 4% paraformaldehyde overnight and embedded in paraffin. The tissue was cut into 5 μm slices, which were stained with H&E to evaluate the severity of lung inflammation, PAS to assess mucus secretion, and Picrosirius red (Sirius red) to visualize collagen deposition or fibrosis. Images were captured with a ZEISS Axio Examiner microscope.

### Immunohistochemistry

The paraffin-embedded tissue was cut into 5 μm sections for IHC analysis. After deparaffinizing and antigen retrieval, the tissues were blocked with 10% goat serum and then incubated with mouse E-cadherin antibody (1:1,000, Cell Signaling Technology, 14472) at 4°C overnight followed by a goat anti-mouse antibody (1:2,000, Abcam, ab205719) for 1 hour at room temperature. Tissue sections were then probed with 3,3-diaminobenzidine as a color developer and counterstained with hematoxylin. Images were captured with a ZEISS Axio Examiner microscope.

### ELISA

ELISA was performed according to the manufacturer’s instructions provided by commercially available ELISA kits. Levels of PYCR1 were measured by an ELISA kit as described before. Total IgE in serum was detected using an ELISA kit (BioLegend, 432404). The levels of cytokines in the lung homogenates, such as IL-4, IL-5, IFN-γ (BioLegend, 431105, 431204, 430804), TGF-β1 (Thermo Fisher Scientific, 88-8350-88), collagen I (Elabscience, E-EL-M0325c) and IL-13 (PeproTech, 900-K207), were measured using commercial ELISA kits as instructed.

### Western blot assay

To measure the expression levels of E-cadherin, Snail, α-SMA, PYCR1, p-AKT, p-Raptor, β-catenin, and WNT3a, Western blotting was performed as previously described. Total proteins in cells or tissues were lysed or homogenized in RIPA buffer (Thermo Fisher Scientific, 89900) supplemented with PMSF solution (Beyotime, ST507-10ml). Cell lysates or lung homogenates were electrophoresed, transferred to a polyvinylidene difluoride membrane, blocked in 5% (*w/v*) nonfat milk, and then probed using their respective antibodies at 4°C. The sources of primary antibodies were E-cadherin (Cell Signaling Technology, 14472), Snail (Cell Signaling Technology, 3879), α-SMA (Abcam, ab5694), PYCR1 (Abcam, ab94780), p-AKT (Cell Signaling Technology, 4060), p-Raptor (Cell Signaling Technology, 89146), β-catenin (Santa Cruz Biotechnology, sc-7963), WNT3a (Santa Cruz Biotechnology, sc-136163), GAPDH (Cell Signaling Technology, 2118), and β-actin (Cell Signaling Technology, 4970). HRP-linked anti-rabbit IgG (Cell Signaling Technology, 7074) or HRP-linked anti-mouse IgG (Cell Signaling Technology, 7076) was used to detect the binding antibody for 1 hour at room temperature. The specific antibody-bound bands were visualized using the gel doc system (Tanon, 5200), and densitometry was performed with ImageJ (NIH).

### Immunofluorescence

For immunofluorescence, cells or slides were fixed (4% paraformaldehyde), permeabilized (0.5% Triton X-100), blocked (5% goat serum), and then stained with anti-PYCR1 (1:200, Proteintech, 13108-1-AP), anti-keratin (1:200, Cell Signaling Technology, 4545), anti–pro–surfactant protein C (anti–pro-SPC; 1:250, Abcam, ab90716), and anti–α-SMA (1:500, Servicebio, GB111364) primary antibodies overnight at 4°C. Afterward, samples were probed with secondary goat anti-rabbit or goat anti-mouse IgG antibody conjugated with Alexa Fluor 555 (Invitrogen, A-21428, A-21422) for 1 hour at room temperature in the dark. Nuclei were stained with DAPI (Yeason, 36308ES20). Images were visualized with a fluorescence microscope (Olympus, model IX73) and analyzed via ImageJ.

### Cell culture and stimulation

MLE-12 cells (CRL-2110, ATCC), a murine bronchial alveolar cell line, were cultured in complete DMEM. BEAS-2B cells (purchased from Procell Life Science & Technology Co. Ltd), a kind of human bronchial epithelium, were cultured in complete bronchial epithelial cell growth medium (Lonza, CC-3170 and CC-4175). The cells were incubated in a humidified atmosphere containing 95% O_2_ and 5% CO_2_ at 37°C. The cells were seeded in 24-well plates overnight and then stimulated with PBS or HDM at doses of 100 μg/mL for 24 hours.

### Isolation and identification of primary tracheal epithelial cells

Primary murine tracheal epithelial cells were isolated and identified as previously described (67–69). Tracheas from control and asthmatic mice were dissected and cut longitudinally in Ham’s F12 (Pricella, PM150810) plus 100 U/mL penicillin and 100 μg/mL streptomycin on ice. Each trachea was individually immersed in 5 mL Ham’s F12/Pen-Strep containing 1.5 mg/mL pronase (Roche, 10165921001) and incubated at 4°C overnight (18–24 hours). Then 500 μL of warmed FBS was added and inverted gently 20 times to inactivate pronase and dislodge cells. The tracheas were removed twice from the medium and immersed in a new 15 mL tube containing 3 mL Ham’s F12/Pen-Strep/10% FBS, inverted 20 times, and finally discarded. Media from the 3 tubes were combined, and cells were pelleted by centrifuging at 500*g* for 10 minutes at 4°C. Cells were then resuspended in 0.5 mg/mL DNase (MilliporeSigma, 10104159001) solution, incubated on ice for 5 minutes, and centrifuged at 500*g* for 10 minutes at 4°C. Cells were resuspended in DMEM/F12/Pen-Strep/10% FBS (mTEC/Basic medium), placed in Primaria tissue culture dishes (Corning, 353803), and incubated at 37°C and 5% CO_2_ for 3–4 hours to attach fibroblasts. The supernatant was collected in a sterile tube. The dish was rinsed twice with warm mTEC/Basic medium/10% FBS, and the medium was pooled in the same collection tube. Epithelial cells were pelleted at 500*g* for 5 minutes at 4°C, and the cells were resuspended in warm bronchial epithelial cell medium (ScienCell Research Laboratories, 3211) (mTEC/Plus medium). Then 10 μL of the cell suspension was mixed with 0.4% trypan blue to count the cells and cell viability.

The average yield of each trachea was 1 × 10^5^ to 2 × 10^5^ cells, and the cell viability was greater than 90%. These cells revealed that more than 90% expressed cytokeratin, as validated by immunofluorescence of pankeratin antibody staining above. Primary airway epithelial cells from control mice and asthmatic mice were lysed using RIPA and PMSF and protease inhibitors to isolate proteins from cells.

### Isolation and identification of primary alveolar epithelial cells

Primary murine alveolar epithelial cells were isolated as previously described (70). Briefly, lungs obtained from 6- to 8-week-old male C57BL/6 mice were anesthetized and perfused with 10–20 mL of PBS into the right ventricle until the lungs were flushed of blood and were white in appearance. The tissue was minced into very small pieces using surgical scissors. The samples were digested with 0.1% collagenase, 0.25% trypsin, and 5 mg/mL DNase I for 45 minutes at room temperature (RT) and were filtered through a 40 μm filter. Subsequently, the suspension containing alveolar epithelium was purified via adherence to IgG-coated plates before 24 hours. The isolated alveolar epithelial cells were cultured in complete DMEM on 24-well plates (1 × 10^6^ cells per well) overnight for adherence. Afterward, the culture media were refreshed and treated with PBS or HDM for 24 hours.

For the identification of primary alveolar epithelial cells, cellular immunofluorescence of pro-SPC, a marker of alveolar epithelial cells, was performed as described above (Supplemental Figure 3).

### Transmission electron microscopy

Transmission electron microscopy was performed under a standard protocol as previously described (71, 72). In brief, tracheas were dissected, and lung tissues were obtained from WT mice and *Pycr1*-deficient mice that were exposed to HDM or PBS for 5 weeks. Fresh tissues (approximately 1 mm × 1 mm × 1 mm in size) from the same location of the lung were quickly soaked in 2.5% glutaraldehyde in 0.1 M phosphoric buffer (pH: 7.4) for 24 hours and postfixed in 1% osmium tetroxide for 2 hours at RT in the dark. After removal and rinsing, the samples were dehydrated with gradient alcohol, infiltrated, and embedded using an Eponate 12 Kit with DMP-30 (TED PELLA Inc, 18010). The blocks were cut into 100 nm–thick sections on an ultramicrotome, stained with lead citrate (2.6%) to prevent CO_2_ staining, and counterstained with uranium acetate. The copper grids were observed under a Hitachi transmission electron microscope (JEM-1400Flash).

### Mitochondrial ECAR measurement

Primary airway epithelial cells were isolated from WT mice and *Pycr1*-deficient mice that were exposed to HDMs or PBS for 5 weeks and then seeded on XFe96 plates (Agilent) at a density of 10,000 cells/well in complete DMEM overnight. The ECAR was measured with a Glycolysis Stress Test Kit according to the manufacturer’s protocol. Reagents were injected as follows: 10 mM glucose at 20 minutes, 1.0 μM oligomycin at 50 minutes, and 50 mM 2-deoxyglucose at 80 minutes. Glucose was injected to test basal glycolysis. Oligomycin, an inhibitor of ATP synthase, was loaded to induce maximal glycolytic metabolism. The ECAR calculated from the proton production rate indirectly indicated basal glycolysis and glycolytic capacity.

### Statistics

Data were summarized using basic descriptive statistics. For continuous variables, data are presented as the means ± SD or means ± SEMs if they met a normal distribution or medians (25%–75% IQR) if they did not. For categorical variables, data are presented as numbers (percentages). For comparisons between 2 groups, continuous variables were analyzed using 2-tailed Student’s *t* test or nonparametric tests, and categorical variables were tested by the χ^2^ test, Fisher’s exact test, or Mann-Whitney *U* test, as appropriate. For comparisons among multiple groups, parametric 1-way ANOVA with Dunnett’s adjustment or Bonferroni’s post hoc test or the nonparametric Kruskal-Wallis test were employed depending on the distribution of the data. For pulmonary function test data, 2-way ANOVA was applied followed by Bonferroni’s post hoc test. ROC curves were generated to assess the predictive ability of the metabolites and related enzymes to correctly identify the prevalence of asthma. Measurement of differential levels of metabolites and abundance and pathway analysis of metabolic profiling were carried out with R software as described above. Other statistical analyses were conducted using GraphPad Prism version 8.0. Variables with a 2-sided *P* < 0.05 were accepted as statistically significant.

### Study approval

Before data and sample collection, all participants provided written informed consent. The study complied with the Declaration of Helsinki and was approved by the Ethics Committee of the First Affiliated Hospital of Nanjing Medical University (Nanjing, China; approval 2019-SR-129). All animal work was approved by the Institutional Animal Care and Use Committee at Nanjing Medical University (Nanjing, China, approval number: IACUC-2007017) and was conducted in accordance with institutional guidelines and regulations.

### Data availability

The data sets used or analyzed in the study are available from the Supporting Data Values XLS file.

## Author contributions

TX, Z Wu, QY, XZ, and YL were responsible for methodology, investigation, review and editing, original draft writing, and formal analysis. CW, MS, JW, JJ, Z Wang, and ZC were responsible for review and editing, methodology, and resources. MZ, MH, and NJ were responsible for conceptualization, supervision, project administration, and funding acquisition. All authors reviewed the final manuscript.

## Supplementary Material

Supplemental data

Supporting data values

## Figures and Tables

**Table 1:**
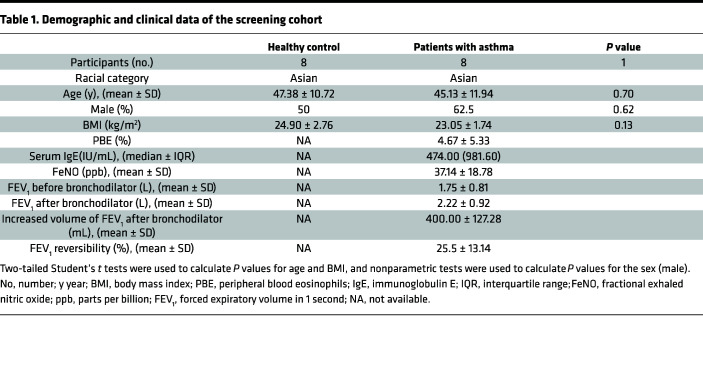
Demographic and clinical data of the screening cohort

**Table 2:**
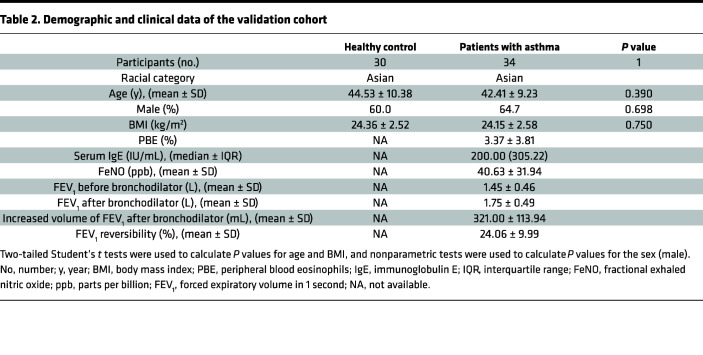
Demographic and clinical data of the validation cohort

**Table 3:**
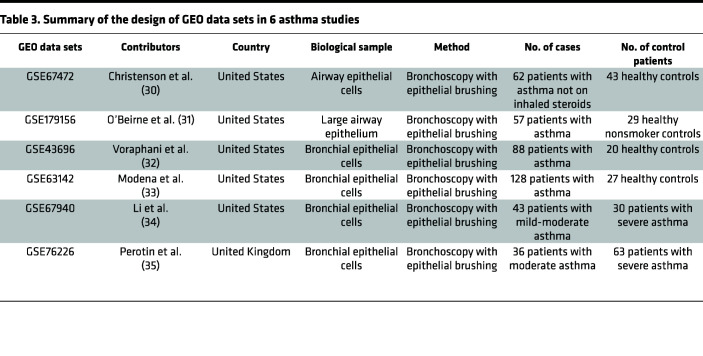
Summary of the design of GEO data sets in 6 asthma studies

## Version 1 07/11/2023

In-Press Preview

## Version 2 08/22/2023

Electronic publication

## References

[1] GBD 2019 Diseases (2020). Global burden of 369 diseases and injuries in 204 countries and territories, 1990-2019: a systematic analysis for the Global Burden of Disease Study 2019. Lancet.

[2] Stolbrink M (2022). The availability, cost, and affordability of essential medicines for asthma and COPD in low-income and middle-income countries: a systematic review. Lancet Glob Health.

[3] Samitas K (2018). Upper and lower airway remodelling mechanisms in asthma, allergic rhinitis and chronic rhinosinusitis: the one airway concept revisited. Allergy.

[4] Meghji J (2021). Improving lung health in low-income and middle-income countries: from challenges to solutions. Lancet.

[5] Mortimer K (2022). Asthma management in low and middle income countries: case for change. Eur Respir J.

[6] Owora AH (2022). Impact of time-varying confounders on the association between early-life allergy sensitization and the risk of current asthma: a post hoc analysis of a birth cohort. Allergy.

[7] Villaseñor A (2017). Allergic asthma: an overview of metabolomic strategies leading to the identification of biomarkers in the field. Clin Exp Allergy.

[8] Heijink IH (2020). Epithelial cell dysfunction, a major driver of asthma development. Allergy.

[9] Hough KP (2020). Airway remodeling in asthma. Front Med (Lausanne).

[10] Miller RL (2021). Advances in asthma: new understandings of asthma’s natural history, risk factors, underlying mechanisms, and clinical management. J Allergy Clin Immunol.

[11] Schoettler N, Strek ME (2020). Recent advances in severe asthma: from phenotypes to personalized medicine. Chest.

[12] Trevor JL, Chipps BE (2018). Severe asthma in primary care: identification and management. Am J Med.

[13] Yang J (2020). Guidelines and definitions for research on epithelial-mesenchymal transition. Nat Rev Mol Cell Biol.

[14] Lee JH, Massagué J (2022). TGF-β in developmental and fibrogenic EMTs. Semin Cancer Biol.

[15] Bakir B (2020). EMT, MET, plasticity, and tumor metastasis. Trends Cell Biol.

[16] Park SY (2022). House dust mite-induced Akt-ERK1/2-C/EBP beta pathway triggers CCL20-mediated inflammation and epithelial-mesenchymal transition for airway remodeling. FASEB J.

[17] Wypych TP (2021). Microbial metabolism of L-tyrosine protects against allergic airway inflammation. Nat Immunol.

[18] Fu L (2022). A mitochondrial STAT3-methionine metabolism axis promotes ILC2-driven allergic lung inflammation. J Allergy Clin Immunol.

[19] Miao Y (2021). The role of GLS1-mediated glutaminolysis/2-HG/H3K4me3 and GSH/ROS signals in Th17 responses counteracted by PPARγ agonists. Theranostics.

[20] Holguin F (2019). L-Citrulline increases nitric oxide and improves control in obese asthmatics. JCI Insight.

[21] Dhodary B (2022). The arginine catabolism-derived amino acid l-ornithine is a chemoattractant for Pseudomonas aeruginosa. Microorganisms.

[22] Liao SY (2020). l-Arginine supplementation in severe asthma. JCI Insight.

[23] Pité H (2018). Metabolomics in asthma: where do we stand?. Curr Opin Pulm Med.

[24] Phang JM (2019). Proline metabolism in cell regulation and cancer biology: recent advances and hypotheses. Antioxid Redox Signal.

[25] Stum MG (2021). Genetic analysis of Pycr1 and Pycr2 in mice. Genetics.

[26] Guo L (2019). Kindlin-2 links mechano-environment to proline synthesis and tumor growth. Nat Commun.

[27] Westbrook RL (2022). Proline synthesis through PYCR1 is required to support cancer cell proliferation and survival in oxygen-limiting conditions. Cell Rep.

[28] Ding Z (2020). Metabolic pathway analyses identify proline biosynthesis pathway as a promoter of liver tumorigenesis. J Hepatol.

[29] He LL (2020). Identification of critical genes associated with the development of asthma by co-expression modules construction. Mol Immunol.

[30] Christenson SA (2015). Asthma-COPD overlap. Clinical relevance of genomic signatures of type 2 inflammation in chronic obstructive pulmonary disease. Am J Respir Crit Care Med.

[31] O’Beirne SL (2021). Up-regulation of ACE2, the SARS-CoV-2 receptor, in asthmatics on maintenance inhaled corticosteroids. Respir Res.

[32] Voraphani N (2014). An airway epithelial iNOS-DUOX2-thyroid peroxidase metabolome drives Th1/Th2 nitrative stress in human severe asthma. Mucosal Immunol.

[33] Modena BD (2014). Gene expression in relation to exhaled nitric oxide identifies novel asthma phenotypes with unique biomolecular pathways. Am J Respir Crit Care Med.

[34] Li X (2015). eQTL of bronchial epithelial cells and bronchial alveolar lavage deciphers GWAS-identified asthma genes. Allergy.

[35] Perotin JM (2019). Epithelial dysregulation in obese severe asthmatics with gastro-oesophageal reflux. Eur Respir J.

[36] Kuo CL (2020). Mitochondrial oxidative stress by Lon-PYCR1 maintains an immunosuppressive tumor microenvironment that promotes cancer progression and metastasis. Cancer Lett.

[37] Sessions DT, Kashatus DF (2021). Mitochondrial dynamics in cancer stem cells. Cell Mol Life Sci.

[38] Ghareghomi S (2021). Fundamental insights into the interaction between telomerase/TERT and intracellular signaling pathways. Biochimie.

[39] Kuruvilla ME (2019). Understanding asthma phenotypes, endotypes, and mechanisms of disease. Clin Rev Allergy Immunol.

[40] Xu S (2022). Metabolomics in asthma: a platform for discovery. Mol Aspects Med.

[41] Liang D (2019). Perturbations of the arginine metabolome following exposures to traffic-related air pollution in a panel of commuters with and without asthma. Environ Int.

[42] Fonseca W (2020). Uric acid pathway activation during respiratory virus infection promotes Th2 immune response via innate cytokine production and ILC2 accumulation. Mucosal Immunol.

[43] Abdulnaby NK (2016). Predictive value of serum uric acid in hospitalized adolescents and adults with acute asthma. Ther Clin Risk Manag.

[44] Cottrill KA (2022). Exacerbation-prone pediatric asthma is associated with arginine, lysine, and methionine pathway alterations. J Allergy Clin Immunol.

[45] Zhou B (2021). Dysregulated arginine metabolism in young patients with chronic persistent asthma and in human bronchial epithelial cells. Nutrients.

[46] Comhair SA (2015). Metabolomic endotype of asthma. J Immunol.

[47] Reinke SN (2017). Metabolomics analysis identifies different metabotypes of asthma severity. Eur Respir J.

[48] Mostaço-Guidolin LB (2019). Defective fibrillar collagen organization by fibroblasts contributes to airway remodeling in asthma. Am J Respir Crit Care Med.

[49] Ojiaku CA (2017). Transforming growth factor β1 function in airway remodeling and hyperresponsiveness. the missing link?. Am J Respir Cell Mol Biol.

[50] Boxall C (2006). The contribution of transforming growth factor-beta and epidermal growth factor signalling to airway remodelling in chronic asthma. Eur Respir J.

[51] Al Amir Dache Z (2020). Blood contains circulating cell-free respiratory competent mitochondria. FASEB J.

[52] Prakash YS (2017). Mitochondrial dysfunction in airway disease. Chest.

[53] Song J (2021). Quality control of the mitochondrial proteome. Nat Rev Mol Cell Biol.

[54] Ma K (2020). Mitophagy, mitochondrial homeostasis, and cell fate. Front Cell Dev Biol.

[55] Szwed A (2021). Regulation and metabolic functions of mTORC1 and mTORC2. Physiol Rev.

[56] Kim J, Guan KL (2019). mTOR as a central hub of nutrient signalling and cell growth. Nat Cell Biol.

[57] Oudaert I (2022). Pyrroline-5-carboxylate reductase 1: a novel target for sensitizing multiple myeloma cells to bortezomib by inhibition of PRAS40-mediated protein synthesis. J Exp Clin Cancer Res.

[58] Milne K (2019). A fragment-like approach to PYCR1 inhibition. Bioorg Med Chem Lett.

[59] Sahu N (2016). Proline starvation induces unresolved ER stress and hinders mTORC1-dependent tumorigenesis. Cell Metab.

[60] Karna E (2020). Proline-dependent regulation of collagen metabolism. Cell Mol Life Sci.

[61] Li Y (2021). PYCR, a key enzyme in proline metabolism, functions in tumorigenesis. Amino Acids.

[62] Reddel HK (2019). GINA 2019: a fundamental change in asthma management: treatment of asthma with short-acting bronchodilators alone is no longer recommended for adults and adolescents. Eur Respir J.

[63] Perez de Souza L (2021). Ultra-high-performance liquid chromatography high-resolution mass spectrometry variants for metabolomics research. Nat Methods.

[64] Zheng F (2020). Development of a plasma pseudotargeted metabolomics method based on ultra-high-performance liquid chromatography-mass spectrometry. Nat Protoc.

[65] Böll S (2020). Acid sphingomyelinase regulates T_H_ 2 cytokine release and bronchial asthma. Allergy.

[66] Kim RY (2017). Role for NLRP3 inflammasome-mediated, IL-1β-dependent responses in severe, steroid-resistant asthma. Am J Respir Crit Care Med.

[67] Horani A (2013). Applications of mouse airway epithelial cell culture for asthma research. Methods Mol Biol.

[68] You Y, Brody SL (2013). Culture and differentiation of mouse tracheal epithelial cells. Methods Mol Biol.

[69] You Y (2002). Growth and differentiation of mouse tracheal epithelial cells: selection of a proliferative population. Am J Physiol Lung Cell Mol Physiol.

[70] Dobbs LG (1986). An improved method for isolating type II cells in high yield and purity. Am Rev Respir Dis.

[71] Gao M (2022). Biomass-related PM2.5 induces mitochondrial fragmentation and dysfunction in human airway epithelial cells. Environ Pollut.

[72] Yao XH (2020). Pathological evidence for residual SARS-CoV-2 in pulmonary tissues of a ready-for-discharge patient. Cell Res.

