# Supplementary material for: Proline is increased in allergic asthma and promotes airway remodeling
Source: JCI Insight. 2023 Aug 22;8(16):e167395. doi: 10.1172/jci.insight.167395 (PMC10543727; doi:10.1172/jci.insight.167395)
Supplement: Supplemental data [file jciinsight-8-167395-s258.pdf]

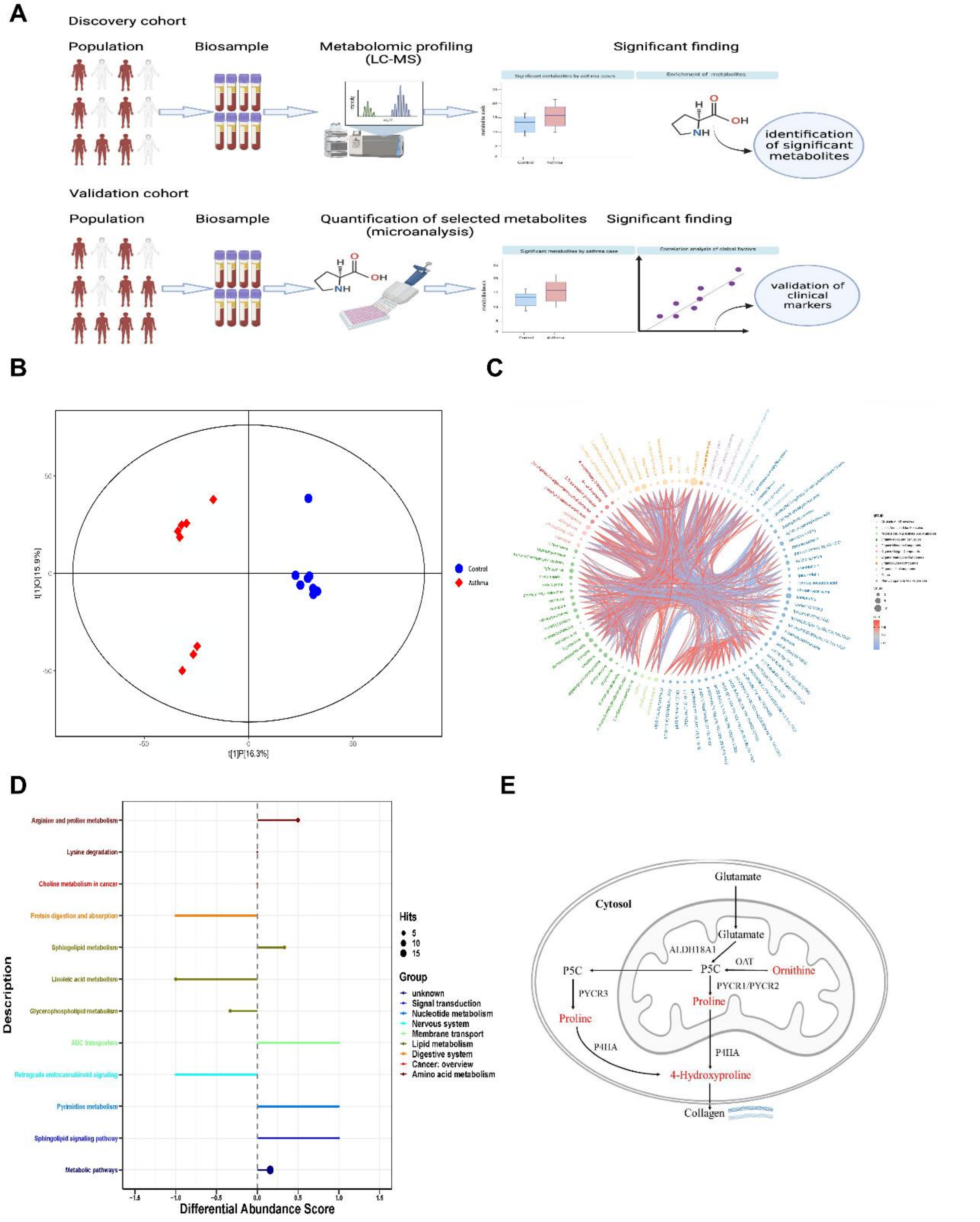

Supplementary Figure 1 Metabolic profiling of plasma reveals altered amino acid levels in asthma.

(A) Schematic diagram of the metabolic profiling protocol for asthmatic patients

and healthy controls. (B) OPLS-DA plot for the assessment of significant metabolites between healthy controls and patients with asthma. (C) Chord diagram for the interrelationships between differential metabolites. (D) Differential abundance score plot for enrichment of metabolites. (E) Schematic depiction of proline biosynthesis and catabolism pathways.

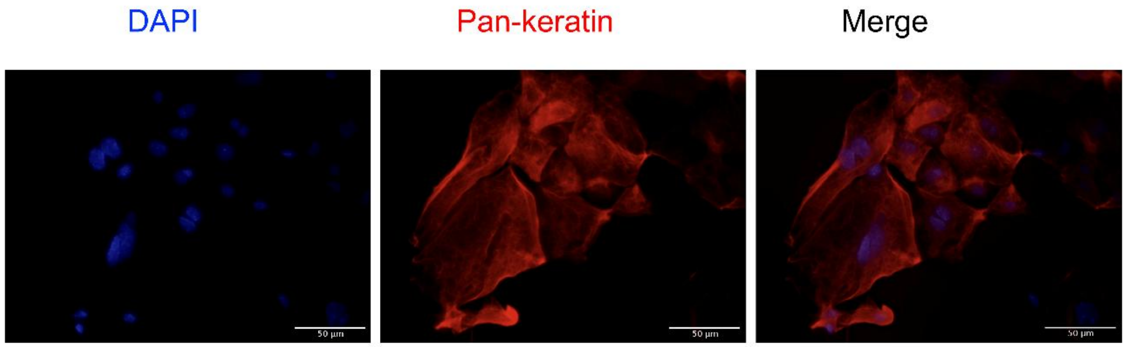

Supplementary Figure 2 Expression of pan-keratin on isolated primary tracheal epithelial cells was determined by immunofluorescence to evaluate the purity of the cells; original magnification 400× (top); scale bar, 50 μm

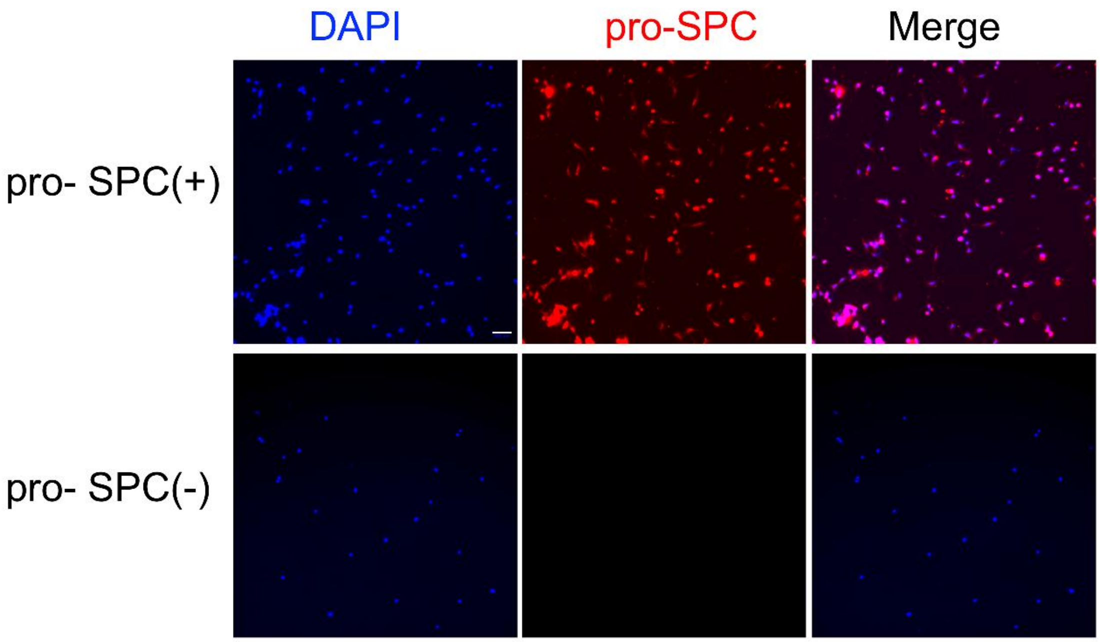

Supplementary Figure 3 Expression of pro-SPC on isolated primary alveolar epithelial cells was determined by immunofluorescence to evaluate the purity of the cells; original magnification 100× (top); scale bar, 200 μm.
